# Combination of genetic studies and animal modeling proposes TMPRSS9 as a candidate gene for serum K^+^ variations

**DOI:** 10.1038/s41598-025-11106-7

**Published:** 2025-07-12

**Authors:** Muriel Auberson, Dongmei Wang, Elodie Ehret, Tanguy Corre, Deepika Anand, Asma Mechakra, Olivier Staub, Murielle Bochud, Edith Hummler

**Affiliations:** 1 Department of Biomedical Sciences, University of Lausanne, 27 rue du Bugnon, 1011 Lausanne, Switzerland; 2 Department of Epidemiology and Health Systems (DESS), University Center for General Medicine and Public Health (UNISANTE), Lausanne, Switzerland; 3 Department of Computational Biology, University of Lausanne, Lausanne, Switzerland; 4 Swiss Institute of Bioinformatics, Lausanne, Switzerland; 5 National Center of Competence in Research “Kidney.CH”, Lausanne, Switzerland

**Keywords:** Proteases, Dietary potassium, Sex specificity, Variation in K^+^, Na^+^ handling, Kidney, Genetic association study

## Abstract

A candidate gene association analysis identified *TMPRSS9* as gene for potassium sensitivity in women. To validate this finding, constitutive and conditional *Tmprss9* knockout mice were generated and subjected to dietary K^+^ deprivation and K^+^ loading. Interestingly, mouse renal *Tmprss9* gene expression was similar in both sexes on standard diet but differed when challenged with K^+^-deprivation or -loading in wildtype (WT) mice. Constitutive deficiency of *Tmprss9* was evidenced on a transcriptional level in knockout (KO) mice. Serum Na^+^ levels were lower in male and female KO mice on low K^+^ (LKD), while on high K^+^ (HKD) diet, serum K^+^ only increased in male KO mice. Upon all diet conditions namely standard diet (SD), LKD and HKD the protein abundances of sodium transporting proteins like the sodium-chloride symporter (NCC), alpha and gamma epithelial sodium channel (ENaC) subunits as well as their ratio of cleaved/full length protein and the sodium-hydrogen exchanger 3 (NHE3) were similar in WT and KO mice and/or showed only minor differences. We propose that in human, *TMPRSS9* may function as a sex-specific modifier gene for serum K^+^ handling in women, whereas in mice, male rather than female *Tmprss9* KO retained serum K^+^ on HKD.

**Supplementary Information:**

The online version contains supplementary material available at 10.1038/s41598-025-11106-7.

## Introduction

Hypertension is a highly prevalent global disease and contributes to cardiovascular disease and all-cause mortality^1^. According to the WHO Global Report on Hypertension, worldwide over 1 billion people are affected. Only 54% of affected adults are diagnosed, 42% receive treatment and a mere 21% control their hypertension^1^. Thereby, genetic and environmental factors as well as individual and sex-specific susceptibility to hypertension must be considered^2^. Increased dietary K^+^ intake reduced blood pressure in hypertensive subjects and attenuated the effect of dietary Na^+^ on vascular function in salt-resistant adults^3^^[,4^. Many transport functions in renal tubules depend on K^+^ channels and affect maintenance of external K^+^ balance and regulation of cell volume^5^. Furthermore, renal K^+^ channels generate a cell-negative electrical potential that affects transmembrane movement of many charged solutes and, the recycling of K^+^ in the thick ascending limb (TAL) plays an important role in the control of NaCl reabsorption^6^. A collecting duct-specific deletion of the K^+^ channel Kcnj10 (Kir4.1) predisposed for thiazide-and low K^+^ diet-induced hypokalemia^7^. Moreover, Kelly and colleagues (2010) looked for loci highly associated with blood pressure response towards a high potassium intake. They used GWAS in a Chinese cohort of healthy volunteers and identified the 11q23.3 locus that included amongst other genes the serine protease *TMPRSS4*^8^. Interestingly, upon K^+^ depletion, KO male but not female mice showed a decreased Na^+^ excretion and developed a dysregulated renal water handling^9^. Not surprisingly, a sexual dimorphic pattern of renal transporters and electrolyte homeostasis was documented in rats that suggested lower proximal Na^+^ transport in females associated with higher distal Na^+^ transporter abundance. This facilitated K^+^ secretion and lowered the serum K^+^ concentration setpoint^10^ altering thereby the renal tubule organization and changing the fractional absorption and/or excretion of ions^11^.

The membrane-bound serine protease *TMPRSS9* (Transmembrane protease, serine 9) was identified as polyprotein also called polyserase-I (polyserine protease-I) with unique three tandem serine protease domains and, is expressed in fetal and adult tissues and tumor cell lines^12^. It is a member of the type II transmembrane serine protease (TTSPs) family^13^. Tmprss9 can be cleaved by trypsin and is inhibited by several natural serine protease inhibitors. The splice variant Serase-1B efficiently converted pro-urokinase-type plasminogen activator (pro-uPA) into active uPA indicating a role in fibrinolysis and tumour progression^14^. Its physiological role in the kidney is however still largely unknown. Furthermore, *TMPRSS9* was found associated with neuroticism, a personality trait associated with negative emotions^15^^[,16^ and, a combination of whole exome sequencing and animal modelling identified recently *TMPRSS9* as a candidate gene for autism spectrum disorder^17^.

To identify loci responsible for K^+^ variations in males and females (linked to blood pressure), we performed a genetic association analysis that identified *TMPRSS9* as a highly significant locus for serum K^+^ variation in women. To further study whether *TMPRSS9* deficiency affected K^+^ balance, we generated a constitutive and conditional KO in mice which were then challenged with K^+^-depletion and/or -loading. Male *Tmprss9* KO mice retained serum K^+^ on HKD, whereas female and male KO mice exhibited decreased serum Na^+^ levels on a LKD. We propose that *TMPRSS9* functions as a modifier gene for K^+^ and Na^+^ handling.

## Results

### *TMPRSS9 *was identified as a candidate gene for potassium sensitivity in women

Initially a Genome-Wide-Association-Study (GWAS) was performed to detect a potential unknown locus associated to potassium levels in the Swiss Kidney Project on Genes in Hypertension (SKIPOGH) cohort, including 949 adult participants from the general population with serum potassium measures^18^. A total of 2.5 × 10^6^ genotyped Single Nucleotide Polymorphisms (SNPs) and an additional 4.0 × 10^6^ imputed SNPs were analyzed. However, that GWAS analysis did not yield any result achieving the commonly accepted genome-wide significant threshold of 5 × 10^−8^ (data not shown).

We next performed a candidate gene analysis exploring the genetic associations at loci targeting four selected genes in human. A single gene score was computed using the PASCAL algorithm^19^ to evaluate each gene taking in account each underlying SNP while keeping a low multiple testing burden. The association with the serine protease 3 (*Tmprss3*)^20^ on human chromosome 21 revealed a score of p = 0.02, not reaching the multiple testing corrected threshold of 4.17 × 10^−3^. Only one gene, *TMPRSS9*, showed significant result resisting multiple testing corrections for the combined analysis (4.06 × 10^−5^). However, that association seemed mainly driven by the women-only sub-group (9.96 × 10^−5^), the men showing no statistically significant association after Bonferroni correction (3.60 × 10^−2^). The associated SNPs lie within the gene locus and its 3’ flanking region (Figure S1).

**Fig. 1:**
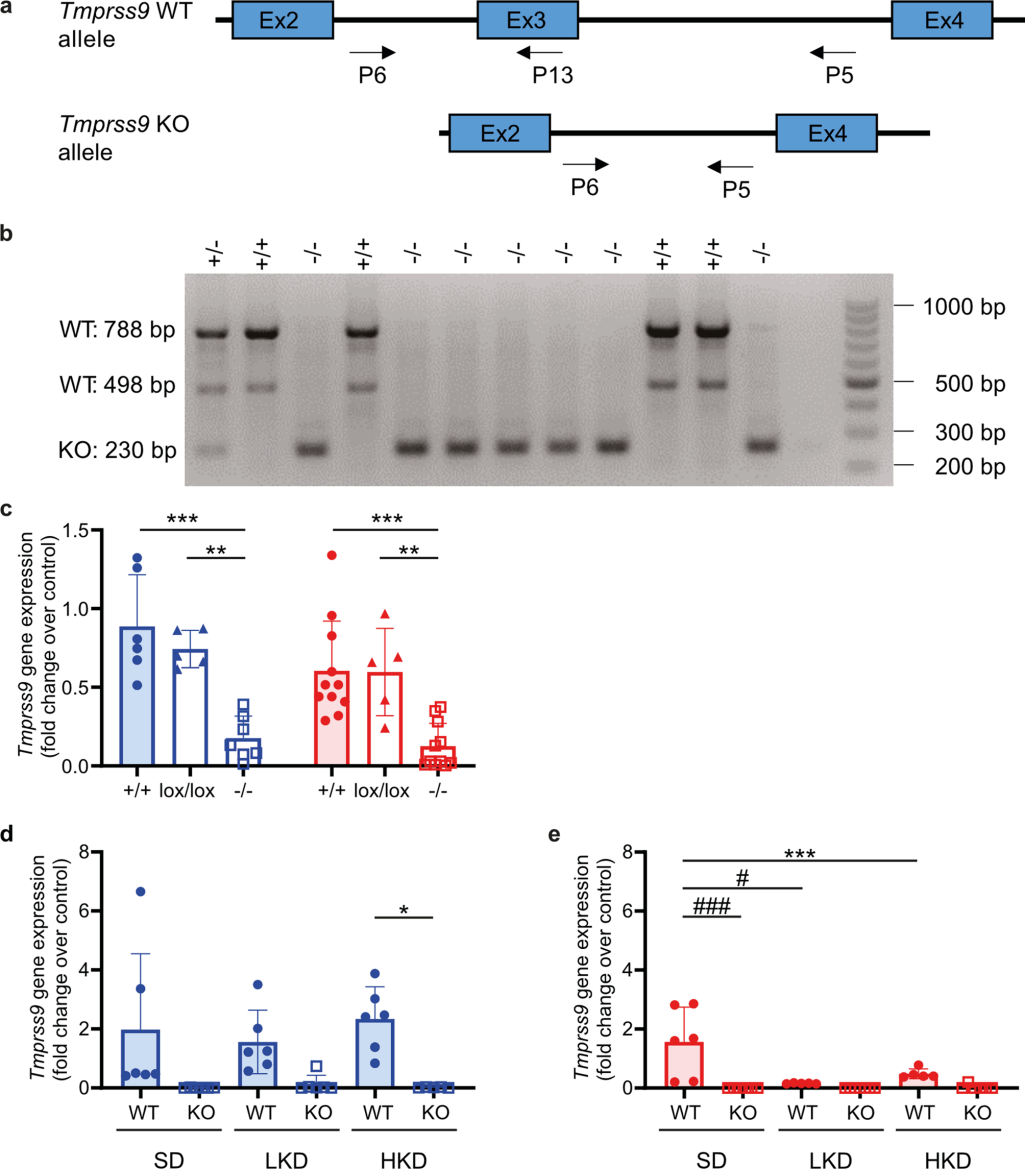
Validation of the conditional and constitutive *Tmprss9* KO mice. (**a**) Scheme of the *Tmprss9* wildtype (WT) and knockout (KO) alleles. (**b**) Genotyping of wildtype (WT, 788 bp and 498 bp), heterozygous mutant (*+/-*, 788 bp, 498 bp and 230 bp) and homozygous mutant (*-/-*, 230 bp) mice in the presence (WT, heterozygote) or absence of exon 3-containing PCR-amplified fragments. (**c**) Spleen mRNA transcript expression of *Tmprss9* in WT (*+/+*, filled circles, *n* = 6–10), floxed (*lox/lox*, triangles, *n* = 5) and KO (*-/-*, open squares, *n* = 7–11). (**d**,** e**) mRNA transcript expression of *Tmprss9* in kidneys from WT and KO on standard (SD), low K^+^ (LKD) or high K^+^ (HKD); blue columns, male; red columns, female mice. Values are mean ± SD. *P* values < 0.05 were considered statistically significant using one-way ANOVA with Tukey’s multiple comparison test; * *P* < 0.05, ** *P* < 0.01, *** *P* < 0.001, difference between genotypes; ^#^ *P* < 0.05, ^###^ *P* < 0.001, difference between diet conditions.

Overall, a significant association to serum potassium variations was identified for the *TMPRSS9* gene locus. That association seems sex-specific for women in a human general population. In mice, *Tmprss9* gene expression was found in spleen of male and female WT and lox/lox mice (Fig. 1c). Additionally, following LKD and HKD, *Tmprss9* mRNA transcript expression in kidney was significantly reduced in female WT mice while no difference in expression was found in males (Fig. 1d, e).

To further validate the implication of this gene in potassium sensitivity, a constitutive (*Tmprss9*^*−/−*^) and a conditional (*Tmprss9*^*lox/lox*^) KO mouse model were generated and metabolically phenotyped (Fig. 1a, b).

### *Tmprss9 *KO mice were viable and male *Tmprss9* KO mice retained K^+^ following high K^+^ challenge

**Fig. 2:**
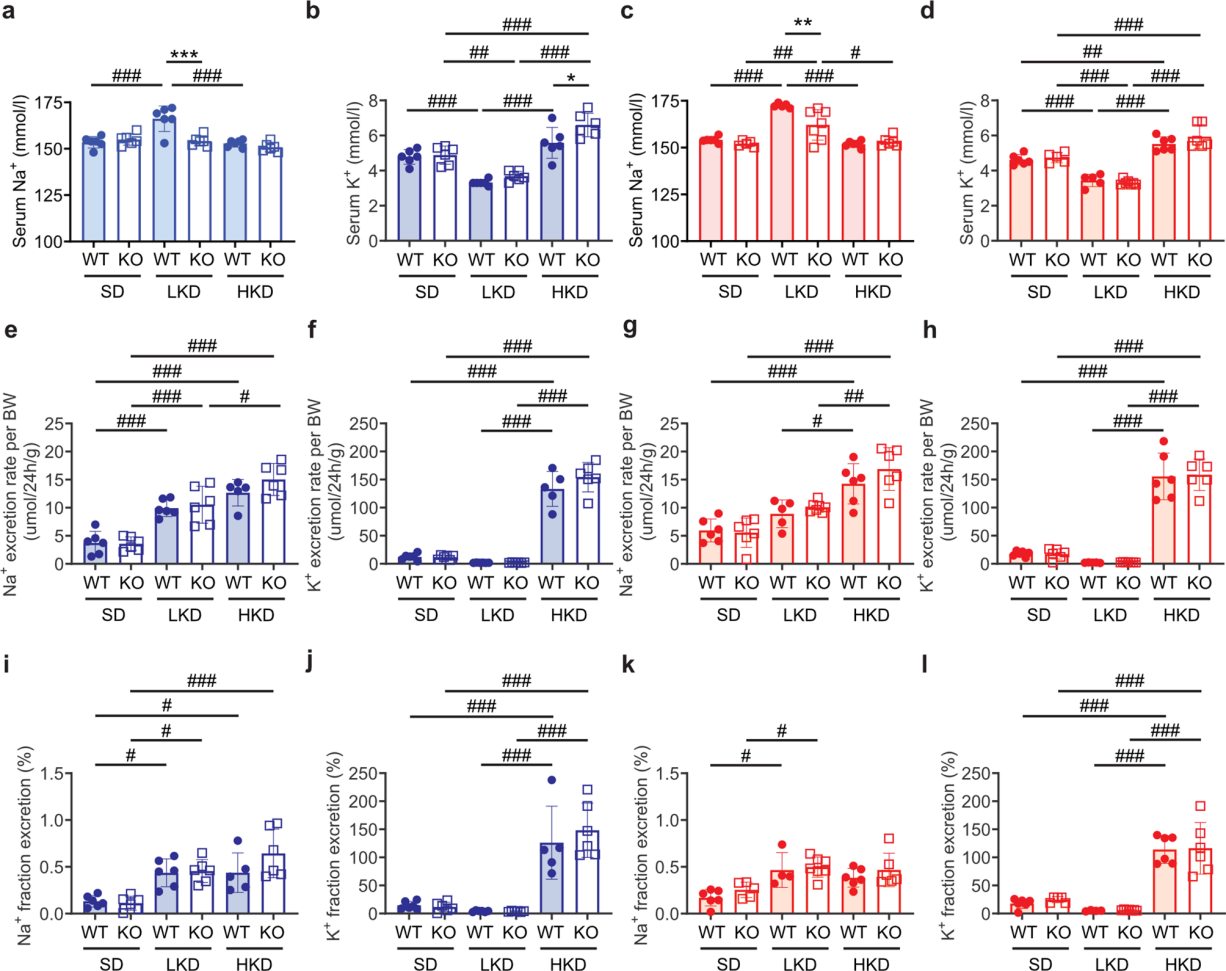
Serum Na^+^ and K^+^ concentrations sex- and diet-specifically differed in *Tmprss9* KO mice. Measurements in male (a, b, e, f, i, j) and female (c, d, g, h, k, l) WT and KO mice of (**a-d**) serum (**a**,** c**) Na^+^ and (**b**,** d**) K^+^ concentrations and, urinary (**e**,** g**) Na^+^ and (**f**,** h**) K^+^ excretion rate. Fractional excretion of (**i**,** k**) Na^+^ and **(j**,** l**) K^+^ in male (blue) and female (red columns) WT (filled circles) and KO (open squares) mice on SD, LKD and HKD. Values are mean ± SD (*n* = 5–7). *P* values < 0.05 were considered statistically significant using one-way ANOVA with Tukey’s multiple comparison test; * *P* < 0.05, ** *P* < 0.01, *** *P* < 0.001, difference between genotypes; ^#^ *P* < 0.05, ^##^ *P* < 0.01, ^###^ *P* < 0.001, difference between diet conditions.

To generate a constitutive and a conditional *Tmprss9* KO, exon 3 was floxed by two *loxP* sites (*Tmprss9*^lox^) using CRISPR-Cas9-mediated gene targeting (Fig. 1a, b; Figure S2). Homozygous mutant male and female floxed *Tmprss9*^lox/lox^ mice exhibited a *Tmprss9* mRNA transcript expression in spleen similar to WT mice (Fig. 1c). Constitutive heterozygous (*Tmprss9*^*+*/−^) and homozygous mutant (*Tmprss9*^−/−^) KO mice were viable and fertile and, quantitative PCR analysis indicated absence of *Tmprss9* mRNA transcript expression in spleen and kidney, respectively (Fig. 1c-e). Whereas the transcriptional expression of *Tmprss9* was unchanged in males, the expression level was decreased in female WT mice on LKD and HKD (Fig. 1d, e). Upon standard diet conditions, all physiological parameters including the initial body weight, food and water intake, urine volume, feces output, blood values and urinary excretion of solutes were similar in WT and KO mice. Sex-specific differences were found between male and female WT mice in body weight, food intake, serum urate, urinary excretion of glucose and protein as well as in the fractional excretion of glucose and protein (Table S1). In the KO group, male and female mice differed in water intake, urinary excretion of protein and fractional excretion of Ca^2+^, urea and protein (Table S1). The LKD resulted in a reduced serum Na^+^ in male and female KO (Fig. 2a-d). The excretion rate of Na^+^ and K^+^ as well as the Na^+^ and K^+^ fractional excretion similarly increased and decreased, respectively diet-dependently in WT and KO of both sexes (Fig. 2e-l). Physiological parameters like body weight, food and water intake, urine volume and feces output as well as serum creatinine or urinary excretion of creatinine did not differ between sexes and genotypes (Table S2). Upon HKD, male KO mice exhibited an increased serum K^+^ concentration compared to WT mice (Fig. 2b**)**, while all other metabolic parameters did not differ (Fig. 2, Table S3). Male KO mice showed an increased Na^+^ excretion rate on HKD compared to LKD (Fig. 2e).

In summary, female and male KO mice exhibited a shift versus decreased serum Na^+^ under LKD and additionally, male KO mice showed an increased serum K^+^ concentration on HKD. We therefore investigated whether this is accompanied by changes in transcriptional and/or translational changes in renal Na^+^ and K^+^ transport systems.

### NCC and ENaC protein abundance were similar in WT and KO mice on SD, LKD and HKD

**Fig. 3:**
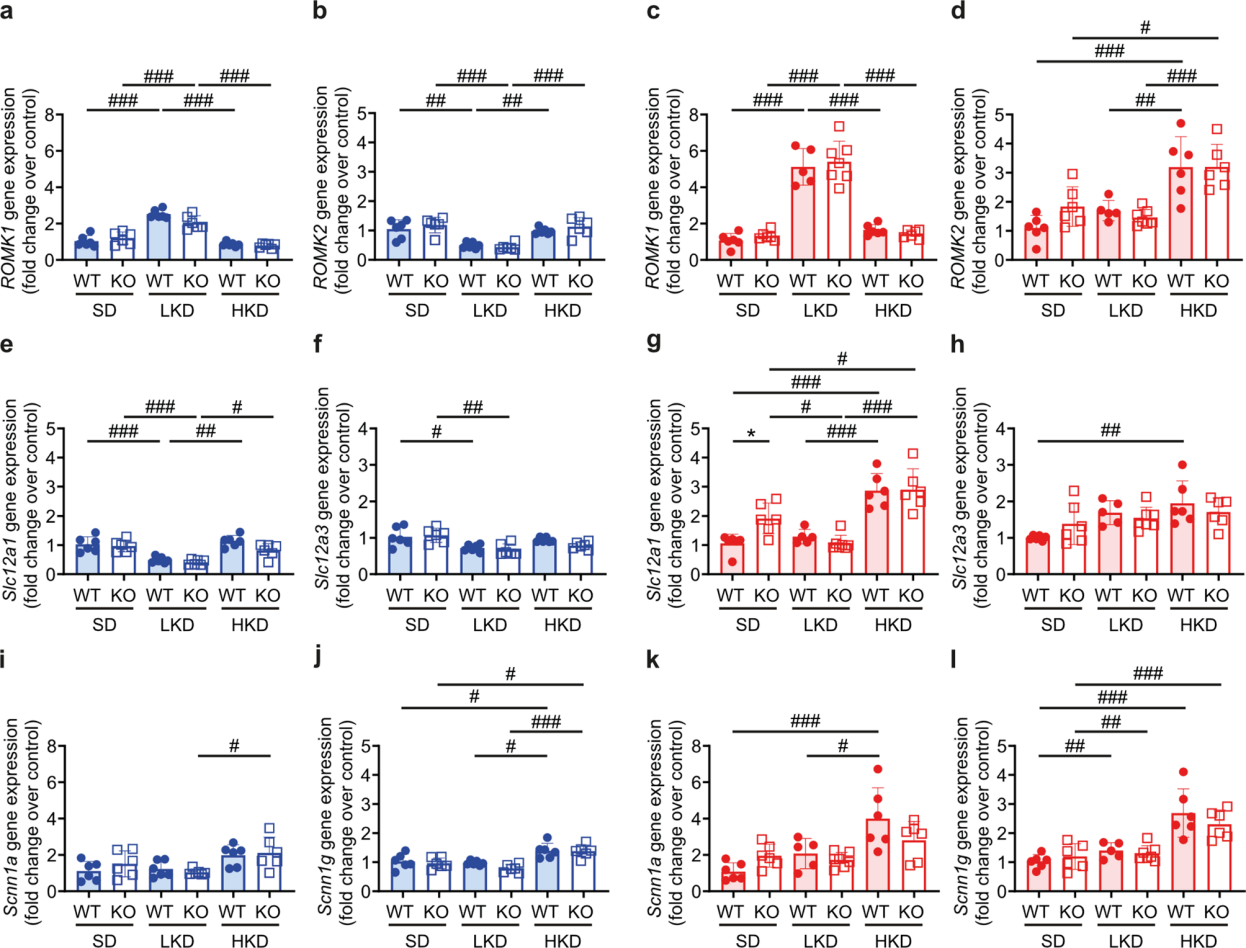
***Slc12a1*** mRNA transcript expression was greater in female *Tmprss9* KO mice on standard diet. (**a**,** c**) Relative mRNA transcript expression of *Romk1*, (**b**,** d**) *Romk2*, (**e**,** g**) *Slc12a1*, (**f**,** h**) *Slc12a3*, (**i**,** k**) *Scnn1a* and (**j**,** l**) *Scnn1g* in kidneys from (a, b, e, f, i, j) male (blue) and (c, d, g, h, k, l) female (red columns) WT (filled circles) and KO (open squares) mice on SD, LKD and HKD. Values are mean ± SD (*n* = 5–7). *P* values < 0.05 were considered statistically significant using one-way ANOVA with Tukey’s multiple comparison test: * *P* < 0.05, difference between genotypes; ^#^ *P* < 0.05, ^##^ *P* < 0.01, ^###^ *P* < 0.001, difference between diet conditions.

Upon all three K^+^ conditions, no difference between female and male WT and KO was found in the gene expression of the renal outer medullary K^+^ channels *Romk1* and *Romk2*, and the α subunit of the epithelium sodium channel (ENaC) *Scnn1a* but this changed following LKD and/or HKD diet (Fig. 3a-d, i-l). In male WT and KO mice, no difference was found in the transcriptional expression of the Na^+^-K^+^-Cl-cotransporter *Slc12a1* and the Na^+^-Cl^−^ cotransporter *Slc12a3* (Fig. 3e, f**)**, whereas in female KO mice, the mRNA transcript expression of the NKCC2 was increased on SD (Fig. 3g, h).

**Fig. 4:**
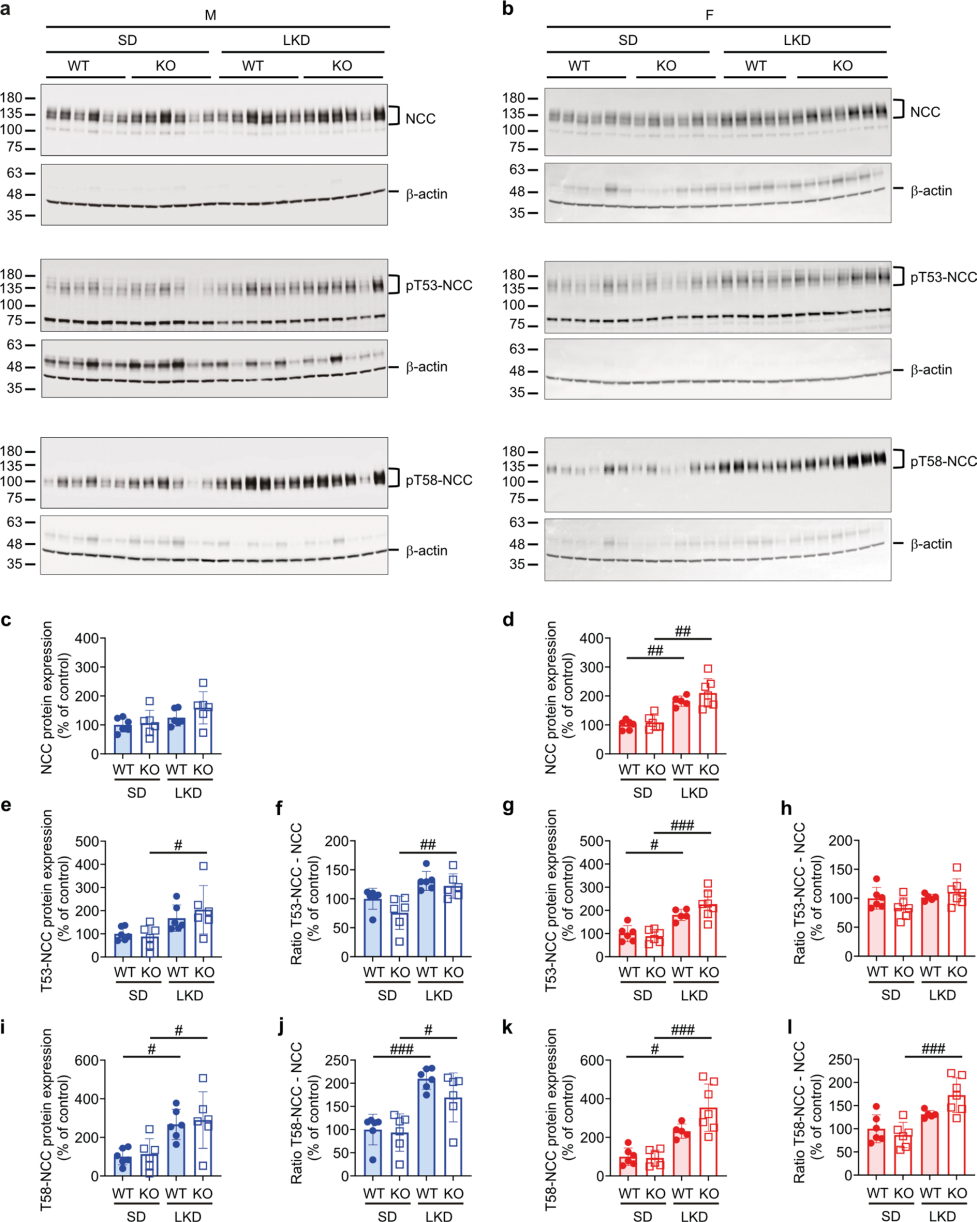
Phosphorylation of NCC was selectively increased in male and female *Tmprss9* KO mice on low K^+^ diet but similar to WT mice. (**a**,** b**) Western blot analysis of NCC, p53-NCC and p58-NCC in kidneys from (**a**) male and (**b**) female WT and KO mice and, (**c-l**) their quantification of male (c, e, f, i, j, blue) and female (d, g, h, k, l, red column) WT (filled circle) and KO (open square) mice on LKD. β-actin was used as loading control. Values are mean ± SD (*n* = 5–7, each group). *P* values < 0.05 were considered statistically significant using one-way ANOVA with Tukey’s multiple comparison test; ^#^ *P* < 0.05, ^##^ *P* < 0.01, ^###^ *P* < 0.001, difference between diet conditions. Uncropped Western blots are presented as Supplementary information.

**Fig. 5:**
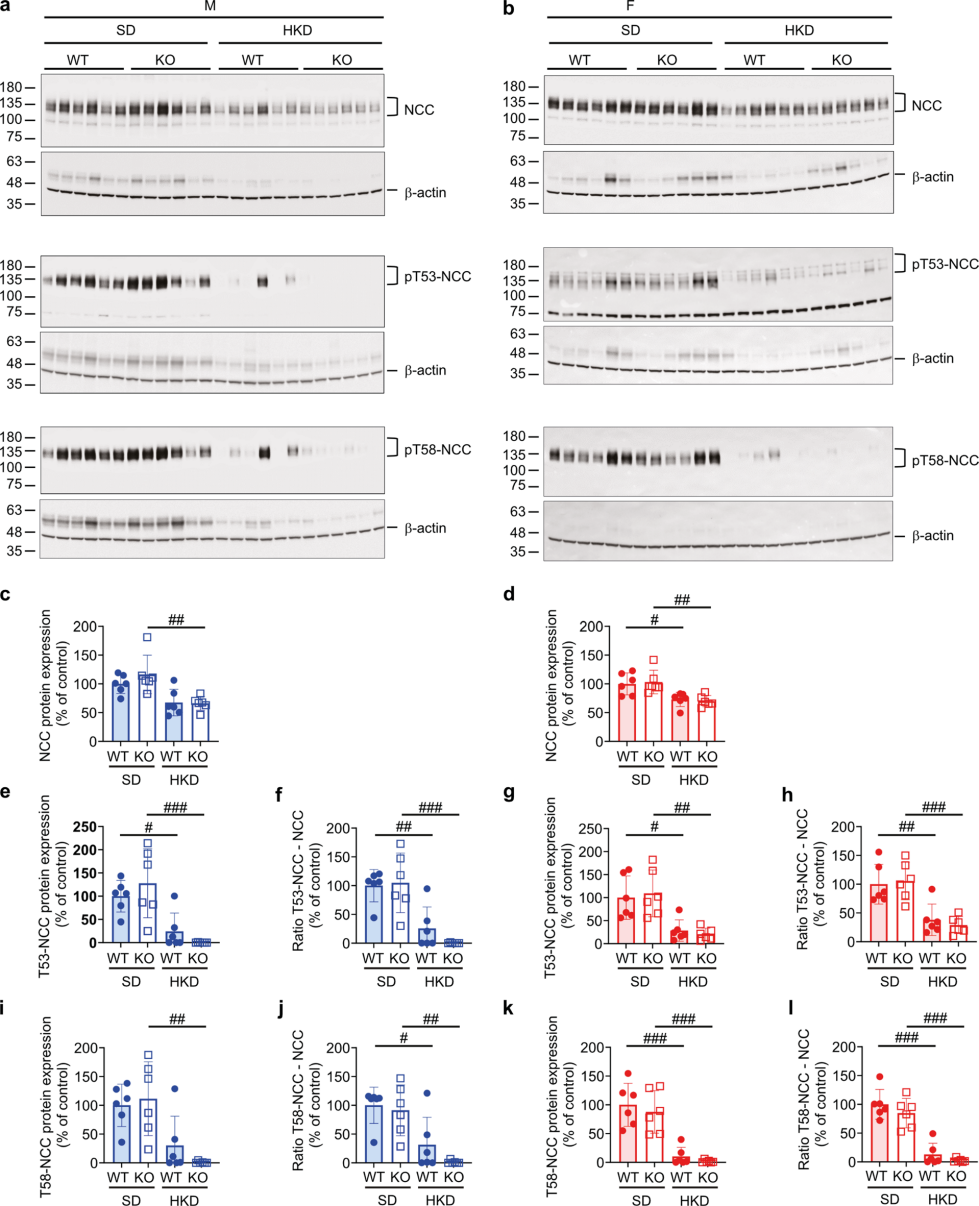
NCC abundance and T58-phosphorylation were similar in WT and KO mice on SD and HKD. (**a**,** b**) Western blot analysis of NCC, p53-NCC and p58-NCCin kidney of (**a**) male and (**b**) female WT and KO and, (**c-l**) their quantification of male (c, e, f, i, j, blue) and female (d, g, h, k, l, red column) WT (filled circle) and KO (open square) mice on SD and HKD. β-actin was used as loading control. Values are mean ± SD (*n* = 6, each group). *P* values < 0.05 were considered statistically significant using one-way ANOVA with Tukey’s multiple comparison test; ^#^ *P* < 0.05, ^##^ *P* < 0.01, ^###^ *P* < 0.001, difference between diet conditions. Uncropped Western blots are presented as Supplementary information.

On a translational level, NCC abundances did not differ in males on SD compared to LKD (Fig. 4a, c). Only the male (Fig. 4a, e) but not female (Fig. 4b, g) KO mice showed an increased T53-NCC phosphorylation and activity measured as T53-phosporylation-to-full-length NCC ratio (Fig. 4f, h) on LKD compared to SD, whereas phospho-T58-NCC abundance was similarly increased in WT and KO groups. This resulted in an augmented T58-NCC activity on LKD (Fig. 4i, j). Similarly, both female WT and KO mice increased NCC abundances on LKD compared to SD (Fig. 4d). T53- and T58-NCC phosphorylation was augmented in both female WT and KO mice on LKD that only increased the T58-NCC activity measured as T58/NCC ratio in KO on LKD (Fig. 4g-l). On HKD, male and female WT and KO mice decreased NCC protein abundance (Fig. 5a-d). T53- and T58-phosphorylation of NCC as well as their ratio of phosphorylated to NCC abundance were similarly decreased in all groups (Fig. 5e-l).

**Fig. 6:**
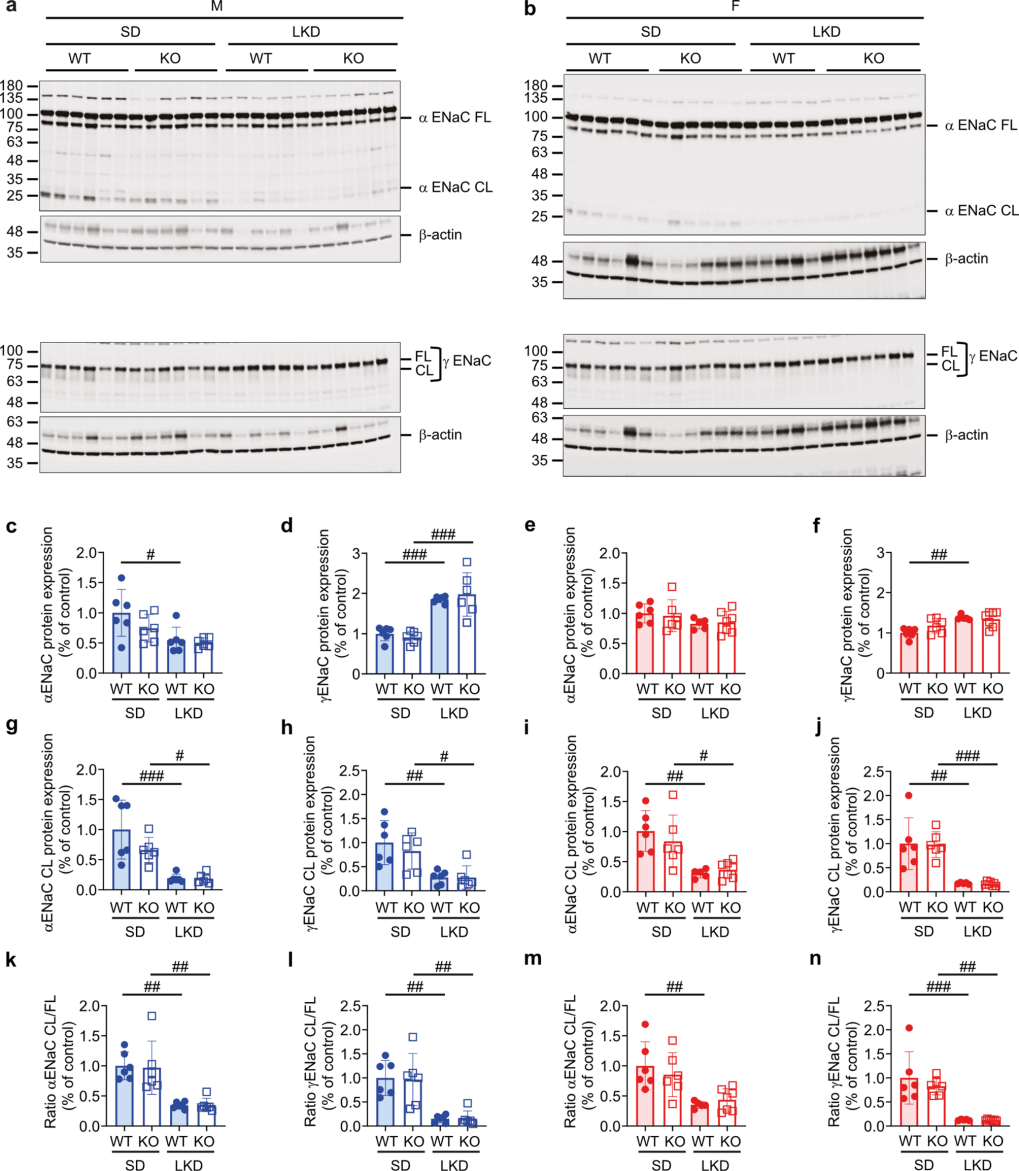
α and γENaC abundance and cleaved/full length protein ratio was similar altered in *Tmprss9* WT and KO mice on LKD. (**a**,** b**) Western blot analysis of αENaC and γENaC in kidney of (**a**) male and (**b**) female WT and KO mice on SD and LKD and, (**c-n**) their quantification (male, c, d, g, h, k, l, blue; female, e, f, i, j, m, n, red column); WT, filled circle; KO, open squares. β-actin was used as loading control; FL, full length; CL, proteolytically cleaved fragment. Values are means ± SD (*n* = 5–7, each group). *P* values < 0.05 were considered statistically significant using one-way ANOVA with Tukey’s multiple comparison test; ^#^ *P* < 0.05, ^##^ *P* < 0.01, ^###^ *P* < 0.001, difference between diet conditions. Uncropped Western blots are presented as Supplementary information.

**Fig. 7:**
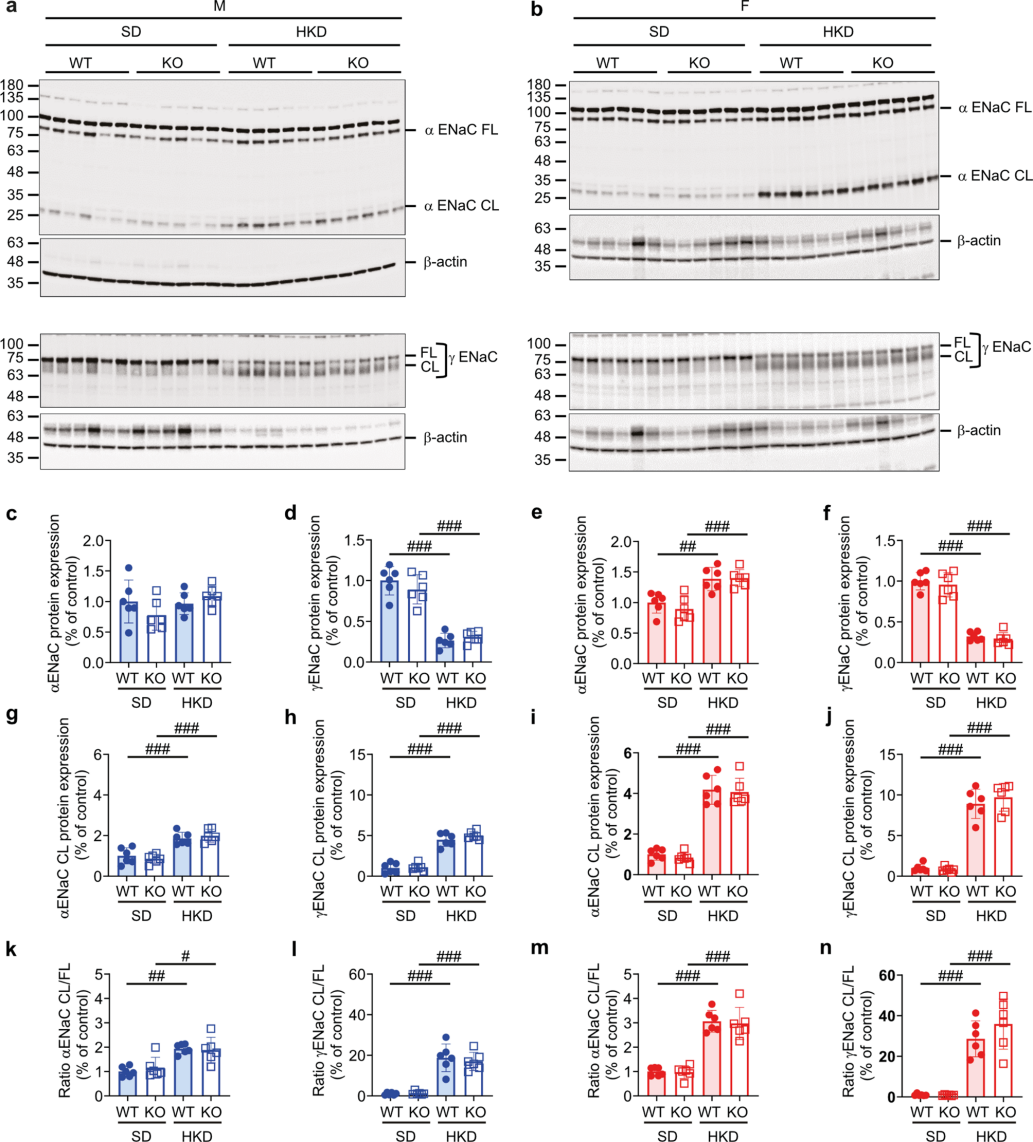
Female WT and KO, but not males increased αENaC protein abundance on HKD. (**a**,** b**) Western blot analysis of αENaC and γENaC in kidney of (**a**) male and (**b**) female WT and KO on SD, HKD and, (**c-n**) their quantification (male, c, d, g, h, k, l, blue; female, e, f, i, j, m, n, red column); WT, filled circle; KO, open squares. β-actin was used as loading control; FL, full length; CL, proteolytically cleaved fragment. Values are mean ± SD (*n* = 5–7). *P* values < 0.05 were considered statistically significant using one-way ANOVA with Tukey’s multiple comparison test; ^#^ *P* < 0.05, ^##^ *P* < 0.01, ^###^ *P* < 0.001, difference between diet conditions. Uncropped Western blots are presented as Supplementary information.

Generally, LKD similarly decreased the full-length and the 25-kDa cleaved αENaC protein abundance in male and female WT and KO mice (Fig. 6a-c, e, g, i), whereas γENaC abundance was rather increased in male and female WT and KO mice (Fig. 6a, b, d, f, h, j). The ratio of α and γENaC cleaved/full length protein abundance was similar in male and female WT and KO mice (Fig. 6k-n). Upon HKD, α and γENaC subunit abundance was similar in all groups and showed no difference between female and male WT and KO mice (Fig. 7a-n).

### Proximal Na^+^ transport and water handling were similar between male and female WT and KO mice

**Fig. 8:**
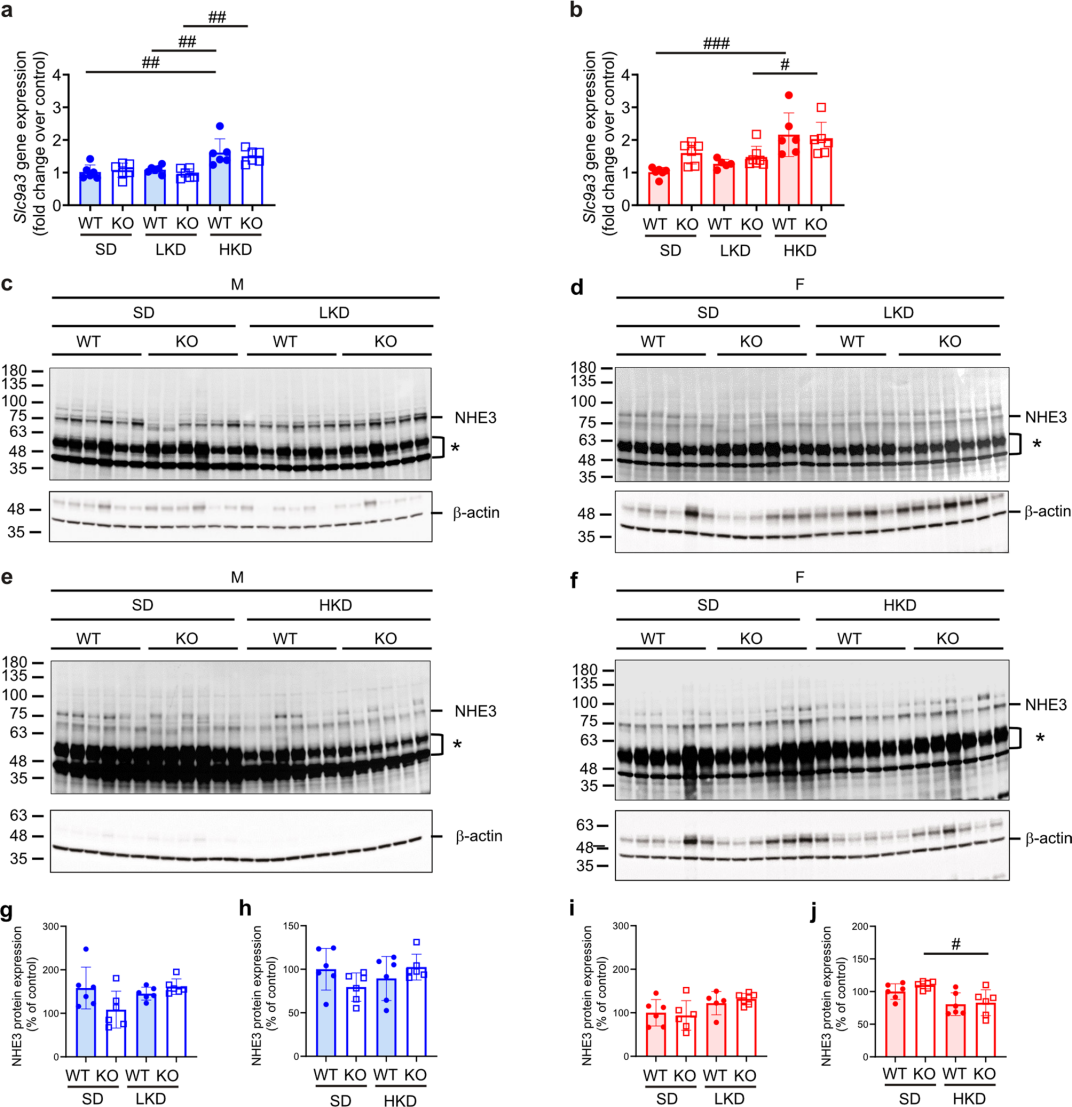
NHE3 protein abundance was similarly decreased in female Tmprss9 WT and KO on high K^+^ diet. (**a**,** b**) Relative mRNA transcript expression of *Slc9a3* in kidneys from (**a**) male (blue) and (**b**) female (red columns) WT (filled circles) and KO (open squares) mice on SD, LKD and HKD. Values are mean ± SD (*n* = 5–7). (**c-f**) Western blot analysis of NHE3 in kidneys from (**c**,** e**) male and (**d**,** f**) female WT and KO mice on LKD (c, d) or HKD (e, g) and, (**g-j**) quantifications are depicted below. β-actin was used as loading control, * non-specific bands. Values are mean ± SD (*n* = 5–7, each group). *P* values < 0.05 were considered statistically significant using one-way ANOVA with Tukey’s multiple comparison test; ^#^ *P* < 0.05, ^##^ *P* < 0.01, ^###^ *P* < 0.001, difference between diet conditions. Uncropped Western blots are presented as Supplementary information.

We next analyzed mRNA transcript and protein expression of the sodium-hydrogen exchanger 3 (NHE3) which is present on the apical side of epithelial cells of the proximal tubule in kidney and, primarily responsible for maintaining the balance of sodium. Upon LKD and HKD, transcriptional (Fig. 8a, b) or translational expression levels (Fig. 8c-j) were similar in all groups. The mRNA transcript expression and protein abundance of the aquaporin 2 channel (Aqp2) was similar in male and female WT and KO mice (Figure S3a-j).

To summarize, both female and male mice exhibited a similar increased and decreased NCC phosphorylation on LKD and HKD, respectively. α and γENaC subunit abundances were diet-specifically altered but did not differ between WT and KO mice of the same sex.

## Discussion

Biological parameters varying between individuals generally have a genetic and heritable component. Although many genes have been discovered, a large fraction of heritability remains unexplained and/or attributable to common genetic variants of smaller impact. A candidate gene association study identified the transmembrane protease serine 9 (*TMPRSS9*) gene locus on chromosome 19 (Fig. S1). Interestingly, the significant association to K^+^ variations was only found in women. A recent study revealed a rising trend of hypokalemia prevalence in the US population being more frequent in women than in men because of their overall lower K^+^ intake. Serum K^+^ levels in men were consistently higher than in women^21^. Hypokalemia is often found in older hypertensive patients treated with K^+^ losing diuretics^22^. Equally, younger persons of both sexes^23^ were prone to hypokalemia due to urinary K^+^ loss likely through a sex-dependent gene reprogramming of the CNT^24^. A sexual dimorphic pattern of renal transporters and electrolyte homeostasis as found in male and female rats at baseline conditions unveiled lower proximal Na^+^ transport. This provoked higher distal Na^+^ transporter abundance that facilitated K^+^ secretion and lowered serum K^+^ set point^10^. Similar abundance profiles were observed in female versus male C57BL/6 mice^10^. In our study, the level of *Tmprss9* mRNA transcript expression showed sexual dimorphism, with females expressing less *Tmprss9* than males on K^+^-deprived and -repleted conditions (Fig. 1c-e). Transcriptional *Tmprss9* expression was particularly low but detectable in many organs, like the kidney and with highest expression in spleen (https://www.proteinatlas.org/ENSG00000178297-TMPRSS9) and cerebellum^17^. RNA-seq analysis of micro-dissected mouse kidney tubule segments unveiled a weak but detectable expression of *Tmprss9* predominantly in the proximal tubules PTS1-PTS3^25^. Deep sequencing in microdissected renal tubules additionally identified low *Tmprss9* expression in the medullary thick ascending limb of the loop of Henle (mTAL) till the outer medullary collecting duct (OMCD)^26^ with slightly increased expression in male mice^27^.

To validate *Tmprss9* as candidate gene for K^+^ variation, we generated constitutive and conditional KO mice targeting exon 3 which should lead to a frame shift and premature stop codon in exon 4 and, thus targeted most if not all splice forms of *Tmprss9.* The floxed *Tmprss9* mice exhibited similar transcriptional expression as WT mice (Figure S2). A *Tmprss9* KO targeting exon 2 recently suggested an association to autism spectrum disorders and, a borderline recognition memory deficit was found in aged female, but not in aged male or younger mice^17^. It was however not excluded that variants including exons 5–12 could be responsible for the mild phenotype^17^. Since we were interested to validate the association of *Tmprss9* in K^+^ handling, male and female control and KO mice were subjected to either K^+^ depletion or K^+^ repletion diet conditions. While no changes were observed on standard conditions, male and female KO mice were not able to adapt their serum Na^+^ levels to LKD, and male KO mice even increased serum K^+^ levels on HKD (Fig. 2a, b).

The major role of the kidney in K^+^ homeostasis depends on its ability to respond effectively to changes in external K^+^ balance and to stabilize its extracellular concentration. The intrinsic ability of distal nephron segments to either secrete or reabsorb K^+^ ensures normal serum K^+^ levels and external K^+^ balance^28^. Thereby, K^+^ secretion is stimulated in principal cells by intake of K^+^ or Na^+^. In animals on a high K^+^ diet, increased activity of apical K^+^ channels was associated with reduced phosphorylation which is reverted on a low K^+^ diet^29^. Whereas K^+^-depletion stimulated K^+^ absorption and blocked Na^+^ transport, chronic K^+^-loading lowered K^+^ and Na^+^ absorption^30^. Both low and high K^+^ intake increased blood pressure and caused Na^+^ retention. Low K^+^ intake was accompanied by an upregulation of the sodium-chloride cotransporter (NCC) and its activating kinase SPAK. High K^+^ intake activated the distal nephron (angiotensin-independent mode of action). High K^+^ intake was associated with elevated serum aldosterone concentrations and an upregulation of the epithelial sodium channel (ENaC) and its activating serum- and glucocorticoid-regulated kinase 1 Sgk1 (aldosterone-independent mechanism). There is functional association of renal K^+^ and Na^+^ handling resulting in Na^+^ retention and high blood pressure when K^+^ intake is either restricted or excessively increased^31^. Vallon and coworkers^32^ reported that mice on low K^+^ diet exhibited increased NCC activity that might decrease Na^+^ and fluid delivery to the distal nephron, thereby negatively affecting K^+^ secretion. In our study and following a LKD, only female KO mice exhibited higher NCC protein abundance and activity and, p58-NCC phosphorylation was additionally increased in female KO vs. WT mice (Fig. 4c, d) suggesting increased susceptibility to K^+^ changes in KO mice. In contrast to the LKD, on a HKD and with the exception of male WT mice, male KO and female WT and KO mice did show a similar decreased NCC p53- and p58-phosphorylation (Fig. 5) which is consistent with previous published data^33^. In a study by Castaneda-Bueno and coworkers, both LKD and HKD were associated with increased phosphorylation in male control mice^34^ while male rats kept 6–8 days on a LKD rather increased, while on a HKD decreased total NCC protein abundance^35^. We could however not exclude species- and/or sex-specific effects as well as varying protocol influencing the outcome. A 6–8 days high (10%) K^+^ diet in male rats resulted in decreased (p44, p48, p53)-NCC phosphorylation^35^. Little and coworkers^36^ reported that NCC levels in mice were lowered following short- and long-term K^+^ supplementation with opposite effects on blood pressure. The study by Welling and coworkers^37^ proposed a K^+^ switch pathway that on low K^+^ diet turns on NCC activity thereby increasing Na^+^ retention, blood pressure and salt-sensitivity. It is however not specified whether males or females were used throughout the study. Sexual dimorphism of renal transporters and electrolyte homeostasis was documented in mice^10^. Females exhibited lower proximal distal Na^+^ transporter abundance to facilitate K^+^ secretion. The distal nephron of female rats showed higher abundances of total and phosphorylated NCC, claudin-7 and cleaved forms of α and γENaC that was associated with lower baseline serum K^+^ concentrations^10^. Indeed, male but not female KO mice showed alterations in serum K^+^ levels following LKD and HKD (Fig. 2b). Interestingly, on HKD only female, but not male mice of both sexes similarly increased full-length αENaC protein abundance (Fig. 7). Female mice might thus be more adapted in conditions of LKD than HKD and might be overall more sensitive to K^+^ diet changes. It may also depend on the experimental conditions such as K^+^ loading and/or exposure time. K^+^ needs to be retained in response to low K^+^ diet and adaptation to gestation^38^. K^+^ balance is regulated by different mechanisms including internal storage or release into or from muscles^39^. Contrary to a previous publication^40^, no significant weight loss was observed in male or female *Tmprss9* WT and KO mice on LKD (Table S2) likely due to its shorter exposure. The amount of the cleaved (presumably active) form of γ ENaC was reduced in all groups on LKD as previously described^41^. The finding that female but not male KO mice did not show increased serum K^+^ on HKD might be explained by undetectable urinary K^+^ loss due to higher volume flow. In human, better adaptation to fluid retention required for pregnancy and lactation has been discussed^42^. Pregnant rats exhibited net K^+^ retention accompanied by markedly increased H^+^-K^+^-ATPase (HKA2) mRNA expression and decreased ROMK- and BK-mediated K^+^ secretion in the kidney^43^. Chronic K^+^ depletion modified the adrenal steroidogenesis and increased progesterone levels that induced HKA2^44^. Interestingly, male but not female *Tmprss4* KO mice displayed altered water handling and urine osmolality, enhanced vasopressin response and cAMP production following a K^+^-deficient diet^9^. The membrane-bound serine protease Tmprss4 was itself upregulated by low dietary K^+^ in distal tubules and its deficiency results in increased renal aquaporin 2 (AQP2) and Na^+^-K^+^−2Cl^−^-cotransporter 2 (NKCC2) expression under K^+^-depletion^9^. However, *Tmprss9* KO mice did not exhibit altered mRNA transcript and protein expression of AQP2 suggesting normal water handling (Figure S3). In this context it is interesting to note that the serine protease *Tmprss9* was identified as a candidate gene for autism spectrum disorder^17^ which comprises a group of neurodevelopmental disorders characterized by impaired social interaction and communication^45^. Over 100 autism spectrum disorder (ASD)-susceptibility genes encoding K^+^ channels have been reported and, any alterations of K^+^ current might change the excitability of neurons and structures of the brain network^17^.

In summary, *TMPRSS9* was identified by gene association analysis in a Swiss cohort population when screening for candidate genes implicated in serum and/or urinary K^+^-variation. *TMPRSS9* was highly associated with changes in serum K^+^ in females. Until now, we have no evidence for a direct or secondary impact of Tmprss9 in the kidney. K^+^-depletion of *Tmprss9* KO mice resulted in mild but significant changes in serum K^+^ levels in males following HKD and, in serum Na^+^ levels of males and females upon LKD. The rather mild K^+^-related phenotype in mice might be explained by the acute (mice) versus more chronic exposure (human) to varying dietary K^+^ concentrations. Further interventional studies in human might reveal its implication in K^+^ and Na^+^ balance. Our findings highly suggested that in human altered *Tmprss9* upon dietary K^+^ challenges might present a confounding factor for the K^+^ and Na^+^ balance.

## Materials and methods

### Genetic association analysis

The whole genome of the SKIPOGH cohort was genotyped before imputation using the HRC r1.1 panel as reference. Associations were carried out by linear regressions correcting for age, center (samples come from three different centers across Switzerland), relationship and additionally by gender for combined analysis.

The PASCAL algorithm gene scoring function was applied to the GWAS results to extract a single association score per gene and per analysis (women only, men only and combined). Considering the 4 genes and the 3 tests per gene (women only, men only, and combined analyses), the Bonferroni threshold was set to 4.174 × 10^−3^ (12 tests).

### Animals and Genotyping

Constitutive mutant (*Tmprss9*^*+/−*^, *Tmprss9*^*−/−*^) and conditional floxed (*Tmprss9*^*floxflox*^) mice were generated by the ETH Phenomics Center (EPIC, ETH Zurich, Switzerland). Briefly, *loxP* sites were inserted 5’ and 3’ to exon 3 of the *Tmprss9* gene using the CRISPR/Cas9-mediated recombination. Deletion of exon 3 led to a frame shift and premature STOP codon in exon 4 resulting in a nonsense-mediated decay of the *Tmprss9* transcript. Correct insertion of the *loxP* sites in floxed *Tmprss9* mice and deletion of 537 bp including exon 3 in constitutive *Tmprss9* KO founders were confirmed by sequencing. Floxed (*Tmprss9*^*lox*/lox^) and knockout (*Tmprss9*^*−/−)*^ mouse lines were kept hereinafter separated. Further detailed information is available on request.

The animal maintenance and the experimental procedures were approved by the Swiss Cantonal and Federal veterinarian authorities (license number VD 3775b). In agreement with the Swiss federal guidelines, the ARRIVE 2.0 recommendations were followed^46^. All experiments were performed in accordance with relevant guidelines and regulations. Mice were housed in a humidity (< 40%) and temperature-controlled room (23 ± 1 °C) with 12 h light/dark cycle (light: 7 am to 7 pm). Mice were kept with free access to food (standard diet, hereinafter referred as normal K^+^ diet, SD, 0.25% Na^+^ and 0.70% K^+^) and tap water in an approved animal care facility of the University of Lausanne. Euthanasia was achieved by deep anesthesia followed by bleeding. Anesthesia was performed by intraperitoneally injecting a mixture of ketamine (80-100 mg/kg body weight) and xylazine (10-15 mg/kg body weight) in PBS till disappearing of the pinch reflex.

Age-matched (3–5 months old, C57BL6/N) male and female homozygous mutant (*Tmprss9*^−/−^, KO) and wildtype (*Tmprss9*^+/+^, WT) littermates were obtained from constitutive heterozygous mutant (*Tmprss9*^+/−^) intercrosses. Conditional heterozygous and homozygous floxed *Tmprss9* mice were obtained by breeding of male and female heterozygous floxed *Tmprss9*^*+/lox*^ mice. All mice were genotyped using PCR-based DNA testing on biopsies using following primers: P2, sense, 5’–GAAACGGATCCCATGTAG–3’; P5, antisense, 5’–TGGGTGGTGGATGGATAGATG–3’; P6, sense, 5’–CATGCCAGCCTGGAATGTG–3’; P13, antisense, 5’–TGCAGAGCATGCGTGAGTAG–3‘ as described previously^47^. Briefly, PCR reactions were carried out on a PeqStar 2x Thermal Cycler (PeqLab Biotechnologie, Erlangen, Germany) using GoTaq DNA Polymerase (Promega Corporation, Madison, WI). PCR protocol was 5 min at 95 °C followed by 35 cycles (1 min at 95 °C, 1 min at 60 °C, and 2 min at 72 °C) and by 10 min at 72 °C. This resulted in following DNA-amplified fragments for the constitutive KO using primers p5, p6 and p13: *Tmprss9*^*+*^ (WT) allele: 498 bp and 788 bp and, the conditional KO using primers p2 and p5: *Tmprss9*^*−*^ (KO) allele: 230 bp; floxed *Tmprss9*^*lox*^ (lox) allele: 438 bp and, (WT) allele: 397 bp.

### Metabolic cages, blood, and urinary analysis

Constitutive male and female *Tmprss9*^*−/−*^ (KO) and *Tmprss9*^*+/+*^ (WT) mice were placed in individual metabolic cages (Tecniplast, Buguggiate, Italy) with free access to food and water for 6 consecutive days. Diet was switched after 2 days from SD to LKD (protocol 1: 0.25% Na^+^, < 0.003% K^+^; 2 days of acclimatization followed by 4 days LKD) or HKD (protocol 2: 0.25% Na^+^, 5% K^+^; 2 days of acclimatization followed by 2 days HKD) diets (ssniff, Spezialdiäten GmbH, Soest, Germany). Body weight, food and water consumption as well as urine and feces output were determined daily. At the end of the experiment, blood was collected and animals sacrified. The 24 h net sodium and potassium excretions (mmol) were calculated by multiplying the concentration (mmol/l) in the collected urine by the urine volume, both collected and assessed during 24 h. The excretion rate was calculated as the concentration of a given substance in the volume of the 24 h urine and the fraction of excretion according the following equation (FE_X_ = (urine [x] × serum [creatinine])/(serum [x] × urine [creatinine]). Serum and urine were analyzed using a Roche/Hitachi 902 robot system (Roche, Mannheim, Germany).

### Real-time PCR

Organs were sampled and homogenized in a TRI Reagent solution (Ambion, Austin, USA) followed by an extraction with 1-bromo-3-chloropropane reagent (BCP, Molecular Research Center, Cincinnati, USA) and an isopropanol precipitation. RNA (1 µg) was reverse transcribed using a PrimeScript RT reagent kit (Takara Biotechnology, Otsu, Japan) according to manufacturer’s guidelines. Real-time PCR was performed using Fast SYBRgreen PCR Master Mix (Thermo Fisher Scientific, Warrington, UK) and run on a QuantStudio 6 Flex Real-Time PCR System (Thermo Fisher Scientific, Warrington, UK). Following primers were used: *Tmprss9*, P3 sense: 5’-GAGCTACATGGGATCCGTTTC-3’, P13 antisense: 5’-TGCAGAGCATGCGTGAGTAG-3’; *Romk1*, sense: 5’-GTGGGCCTCAAAGAAGTCGG-3‘, antisense: 5’-GGAGACCAACCTTGCTCGTT-3‘; *Romk2*, sense: 5’-CCTTTAGCTGGGGCATCCAA-3‘, antisense: 5’-GAGTACGGTTGTCAGGTGGG-3‘; *Slc12a1*, sense: 5’-TTGGATATAACCCACGCCTTTACG-3‘, antisense: 5’-GCCATGCCGCTGTTCATCTC-3’; *NCC*, sense: 5’-CTGGAGAACCTGTTCGCTTC-3’, antisense: 5’-GATGATGAGCCAAGTCAGCA-3’; *Scnn1a*, sense: 5’-AAAGAGAAGCGGGAGTCAGC-3’, antisense: 5’-CGGTGAGTTGGAGACGTCAA-3’; *Scnn1g*, sense: 5’-CCGAGATCGAGACAGCAATGT-3’, antisense: 5’-CGCTCAGCTTGAAGGATTCTG-3’; *Gapdh*, sense: 5’-CCACCCAGAAGACTGTGGAT-3‘, antisense: 5’-CACATTGGGGGTAGGAACAC-3‘. Each measurement was performed in triplicate. The relative expression of each gene was calculated using the comparative 2[−ΔΔCT] method, normalized to *Gapdh*. Data are represented as relative fold-change compared to control mice.

### Immunoblotting

For protein extraction, samples were prepared from organs following one freeze-thaw cycle (−80 °C frozen, single-use aliquot) and then homogenized using TissueLyser (Qiagen, Hilden, Germany) in extraction buffer (Tris pH 7.5 50 mM, EDTA 1 mM, EGTA 1 mM, sucrose 0.27 mM with protease inhibitors complete, Roche, #11836145001 and phosphatase inhibitors phosSTOP, Sigma, #04906837001). After centrifugation at 11`200 rpm for 15 min, supernatant was collected and, total protein concentration was determined using a BCA protein assay kit (Pierce, Rockford, USA). Equal amounts of protein (10 µg) were separated on 4–15% gradient SDS polyacrylamide gels (Bio-Rad, Hertfordshire, UK) and blotted onto nitrocellulose membrane (Whatman, Dassel, Germany). Following primary and secondary antibody exposure (see Table S4) blots were revealed by chemiluminescence (WesternBright Quantum, Witec, Switzerland).

### Statistical analyses

Results are presented as means ± SD. Statistical analyses were performed using GraphPad Prism 10.0 software (GraphPad Software Inc.). One-way ANOVA with Tukey’s multiple comparison test was used for comparison between groups. *P* values < 0.05 were considered as statistically significant.

## Electronic supplementary material

Below is the link to the electronic supplementary material.

Supplementary Material 1

## Data Availability

All data generated and analyzed in this study are available from the corresponding author upon reasonable request.
