# Supplementary material for: Combination of genetic studies and animal modeling proposes TMPRSS9 as a candidate gene for serum K+ variations
Source: Sci Rep. 2025 Jul 12;15:25211. doi: 10.1038/s41598-025-11106-7 (PMC12255782; doi:10.1038/s41598-025-11106-7)

Combination of genetic studies and animal modeling proposes TMPRSS9 as a candidate gene for serum K^+ variations

Muriel Auberson[1], Dongmei Wang[1], Elodie Ehret[1], Tanguy Corre[2,3,4], Deepika Anand[1,5], Asma Mechakra[1], Olivier Staub[1], Murielle Bochud[2,5], Edith Hummler[1,5*]

Supplementary Information

Figure S1. Regional plot displaying association to serum potassium in women at the *Tmprss9* locus on chromosome 19.

Figure S2. Exon 3 of the *Tmprss9* gene locus was targeted by *loxP*-sites.

Figure S3. AQP2 abundances were similar in WT and KO on standard, low and high K^+ diet.

Tables S1-S3. Physiological parameters of WT and *Tmprss9* KO mice on SD, LKD and HKD.

Table S4. Antibodies used for Western blotting

Uncropped Western blots parts 1 to 6

References

Supplementary figure legends

Figure S1 Regional plot displaying association to serum potassium in women at the *Tmprss9* locus on chromosome 19.

Regional association plot showing −log10 (P values) for the association of SNPs at the *Tmprss9* locus ordered by their chromosomal position (and reflecting their physical distance, x-axis) with serum potassium levels. The -log10 (P value) for each SNP is represented by its vertical position according to the scale shown on the left-side y-axis. Each SNP is colored according to the correlation of the corresponding SNP with the SNP showing the lowest P value (rs1050009) within the locus as a reference, using different colors for selected levels of Linkage Disequilibrium (color scale in the box on the left). Correlation structures correspond to HapMap 2 CEU. The blue line represents the recombination according to the scale shown on the right-side y-axis. (plot generated by locus zoom (http://locuszoom.org/)).

Figure S2. Exon 3 of the *Tmprss9* gene locus was targeted by *loxP*-sites.

(**a**) Scheme of the *Tmprss9* WT and floxed gene loci. (**b**) Genotyping of wildtype (WT; 397bp), heterozygous mutant (*lox/+;* 438bp and 397bp) and homozygous mutant (*lox/lox*) mice indicating the presence of *loxP*-sites 5' and 3' of the exon 3.

Figure S3. AQP2 mRNA transcript and protein expression were similar in WT and KO but different in male and female mice on standard, low and high K^+ diet.

(**a,b**) Relative mRNA transcript expression of *Aqp2* in kidneys from (**a**) male (blue) and (**b**) female (red colums) WT (filled circles) and KO (open squares) mice on SD, LKD and HKD. Values are mean ± SD (n= 5-7). (**c-f**) Western blot analysis of AQP2 and beta-actin in kidneys from (**c,e**) male and (**d,f**) female WT and KO mice and, (**g-j**) their quantifications. Beta-actin was used as loading control. Values are mean ± SD (n= 5-7, each group). *P* values < 0.05 were considered statistically significant using one-

way ANOVA with Tukey's multiple comparisons test; # $P < 0.05$, ## $P < 0.01$, ### $P < 0.001$, difference between diet-conditions. Uncropped Western blots are presented as Supplementary information.

Uncropped Western blots.

Part 1 contains the full-length original blots for main Figure 4a-b. Part 2 contains the full-length original blots for main Figure 5a-b. Part 3 contains the full-length original blots for main Figure 6a-b. Part 4 contains the full-length original blots for main Figure 7a-b. Part 5 contains the full-length original blots for main Figure 8c-f. Part 6 contains the full-length original blots for main Figure S3c-f. For each blot, the cropped area is indicated by a black square. Molecular weights are indicated on each blot.

Supplementary Tables

Table S1 Physiological parameters of WT and *Tmprss9* KO mice on standard diet.

	**WT**		***Tmprss9* KO**	
	M	F	M	F
Body weight (g)	29.27 ± 2.78	22.04 ± 3.15$$	29.28 ± 3.12	25.81 ± 3.45
Food intake (g/g BW)	0.13 ± 0.03	0.2 ± 0.03$$	0.12 ± 0.02	0.17 ± 0.04
Water intake (ml/g BW)	0.13 ± 0.03	0.19 ± 0.03	0.09 ± 0.05	0.16 ± 0.05$
Urine volume (ml/g BW)	0.02 ± 0.01	0.03 ± 0.02	0.02 ± 0.01	0.03 ± 0.02
Feces (g/g BW)	0.05 ± 0.01	0.08 ± 0.01	0.05 ± 0.02	0.07 ± 0.03
Blood values				
Ca^{2+}, mM	2.22 ± 0.08	2.29 ± 0.03	2.25 ± 0.05	2.23 ± 0.07
Mg^{2+}, mM	1.09 ± 0.06	1.08 ± 0.07	1.12 ± 0.06	1.09 ± 0.04
Urea, µM	6.75 ± 0.99	7.77 ± 1.35	7.17 ± 1.09	7.13 ± 2.57
Creatinine, µM	9.04 ± 3.48	10.47 ± 2.37	9.41 ± 4.53	12.53 ± 2.48
Urate, µM	136.7 ± 16.1	77.8 ± 15.1$$$	123.6 ± 21.3	101.7 ± 24.0
Glucose, mM	16.22 ± 2.54	12.76 ± 1.85	12.9 ± 2.59	12.22 ± 2.77
Protein, g/l	48.67 ± 2.34	50.38 ± 2.98	50.76 ± 2.1	48.83 ± 2.48
Urinary excretion rate per BW				
Ca^{2+}, µmol/24h/g BW	0.03 ± 0.02	0.06 ± 0.02	0.03 ± 0.01	0.07 ± 0.04
Mg^{2+}, µmol/24h/g BW	0.82 ± 0.39	0.96 ± 0.33	0.88 ± 0.32	1.19 ± 0.63
Creatinine, µmol/24h/g BW	0.16 ± 0.06	0.19 ± 0.06	0.15 ± 0.04	0.18 ± 0.09
Urea, µmol/24h/g BW	0.05 ± 0.03	0.07 ± 0.02	0.05 ± 0.02	0.07 ± 0.03
Urate, µmol/24h/g BW	0.03 ± 0.02	0.03 ± 0.01	0.02 ± 0.02	0.02 ± 0.01
Glucose, µmol/24h/g BW	0.09 ± 0.04	0.2 ± 0.07$	0.09 ± 0.02	0.15 ± 0.09
Protein, mg/24h/g BW	0.26 ± 0.12	0.08 ± 0.03$$	0.17 ± 0.06	0.04 ± 0.02$
Fractional excretion				
Ca^{2+}, %	0.06 ± 0.03	0.15 ± 0.08	0.09 ± 0.04	0.2 ± 0.05$
Mg^{2+}, %	4 ± 0.96	4.77 ± 0.45	4.73 ± 2.23	7.14 ± 1.97
Urea, %	44.21 ± 25.63	56.31 ± 24.96	45.03 ± 26.61	63.9 ± 13.7$$
Urate, %	1.09 ± 0.87	1.98 ± 0.61	1.09 ± 1.11	1.65 ± 1.11
Glucose, %	0.04 ± 0.02	0.09 ± 0.04$	0.05 ± 0.03	0.09 ± 0.02
Protein, %	0.03 ± 0.01	0.01 ± 0$$$	0.02 ± 0.01	0.01 ± 0$

Values are mean ± SD (n = 6). $ difference between males and females. $ *P* < 0.05, $$ *P* < 0.01, $$$ *P* < 0.001 by one-way ANOVA with Tukey's multiple comparisons test.

Table S2 Physiological parameters of *Tmprss9* WT and KO mice following 4 days of low K^+ diet.

	**WT**		***Tmprss9* KO**	
	M	F	M	F
Body weight (g)	27.75 ± 1.68	25.14 ± 1.62	27.35 ± 1.98	22.78 ± 2.58$^{\$\$}$
Food intake (g/g BW)	0.14 ± 0.02	0.14 ± 0.02	0.14 ± 0.02	0.17 ± 0.02
Water intake (ml/g BW)	0.19 ± 0.04	0.2 ± 0.04	0.22 ± 0.07	0.27 ± 0.06
Urine volume (ml/g BW)	0.09 ± 0.02	0.07 ± 0.04	0.12 ± 0.05	0.1 ± 0.04
Feces (g/g BW)	0.02 ± 0.01	0.03 ± 0	0.02 ± 0	0.03 ± 0
Blood values				
Creatinine, µM	11.15 ± 2.78	10.53 ± 1.96	10.8 ± 2.21	13.53 ± 1.99
Urinary excretion rate per BW				
Creatinine, µmol/24h/g BW	0.16 ± 0.02	0.12 ± 0.03	0.16 ± 0.04	0.17 ± 0.03

Values are mean ± SD (n = 5-7). $^{\$}$ difference between males and females. $^{\$\$}$ $P < 0.01$ by one-way ANOVA with Tukey's multiple comparisons test.

Table S3 Physiological parameters of *Tmprss9* WT and KO mice following 2 days of high K^+ diet.

	**WT**		***Tmprss9* KO**	
	M	F	M	F
Body weight (g)	23.43 ± 2.76	19.83 ± 2.13	22.36 ± 2.8	20.5 ± 2.37
Food intake (g/g BW)	0.14 ± 0.03	0.2 ± 0.03$^{\$\$}$	0.16 ± 0.03	0.18 ± 0.02
Water intake (ml/g BW)	0.41 ± 0.15	0.47 ± 0.09	0.38 ± 0.08	0.44 ± 0.12
Urine volume (ml/g BW)	0.2 ± 0.08	0.21 ± 0.07	0.19 ± 0.04	0.19 ± 0.07
Feces (g/g BW)	0.02 ± 0.01	0.04 ± 0.01$^{\$\$}$	0.02 ± 0	0.03 ± 0
Blood values				
Creatinine, µM	11.37 ± 3.05	8.33 ± 2.29	10.47 ± 1.94	10.55 ± 1.69
Urinary excretion rate per BW				
Creatinine, µmol/24h/g BW	0.21 ± 0.03	0.2 ± 0.02	0.17 ± 0.04	0.26 ± 0.05

Values are mean ± SD (n = 6). $^{\$}$ difference between males and females. $^{\$\$}$ $P < 0.01$ by one-way ANOVA with Tukey's multiple comparisons test.

Table S4 Antibodies used for Western blotting

Antibody	Host	Dilution	Source	Reference
NCC	rabbit[a]	1:5,000	J. Loffing	[1]
pT53 NCC	rabbit[a]	1:1,000	J. Loffing	[2]
pT58 NCC	rabbit[a]	1:1,000	J. Loffing	[2]
α-ENaC	rabbit[a]	1:5,000	J. Loffing	[3]
γ-ENaC	rabbit[a]	1:1,000	J. Loffing	[3]
NHE3	mouse[b]	1:1[c]	O. Moe	[4,5]
AQP2	rabbit[a]	1:10,000	J. Loffing	[3]
β-Actin	mouse[b]	1:5,000	Sigma	clone AC-15 (#A1978)

Secondary [a]anti-rabbit, [b]anti-mouse antibodies (1:5000, Amersham Biosciences, Buckinghamshire, UK). [C]Supernatant of hybridoma cells against opossum NHE3

References

1. Loffing, J., Vallon, V., Loffing-Cueni, D., Aregger, F., Richter, K., Pietri, L., Bloch-Faure, M., Hoenderop, J.G., Shull, G.E., Meneton, P. & Kaissling, B. Altered renal distal tubule structure and renal Na(+) and Ca(2+) handling in a mouse model for Gitelman's syndrome. *J. Am. Soc. Nephrol. JASN* 15, 2276–2288 (2004).
2. Sorensen, M. V., Grossmann, S., Roesinger, M., Gresko, N., Todkar, A. P., Barmettler, G., Ziegler, U., Odermatt, A., Loffing-Cueni, D. & Loffing, J. Rapid dephosphorylation of the renal sodium chloride cotransporter in response to oral potassium intake in mice. *Kidney Int.* 83, 811–824 (2013).
3. Wagner, C. A., Loffing-Cueni, D., Yan, Q., Schulz, N., Fakitsas, P., Carrel, M., Wang, T., Verrey, F., Geibel, J. P. Giebisch, G., Hebert, S. C. & Loffing, J. Mouse model of type II Bartter's syndrome. II. Altered expression of renal sodium- and water-transporting proteins. *Am. J. Physiol. Renal Physiol.* 294, F1373-1380 (2008).
4. Bobulescu, I. A., Dwarakanath, V., Zou, L., Zhang, J., Baum, M. & Moe, O. W. Glucocorticoids acutely increase cell surface Na+/H+ exchanger-3 (NHE3) by activation of NHE3 exocytosis. *Am. J. Physiol. Renal Physiol.* 289, F685-691 (2005).

5. Baum, M., Twombley, K., Gattinenio, J., Joseph, C., Wang, L., Zhang, Q., Dwarakanath, V. & Moe, O. W. Proximal tubule Na+/N+ exchanger activity in adult NHE8$^{-/-}$, NHE3$^{-/-}$ and NHE3$^{-/-}$/NHE8$^{-/-}$ mice. *Am J. Physiol. Renal Physiol*. 303, F1495-1502 (2012).

Figure S1

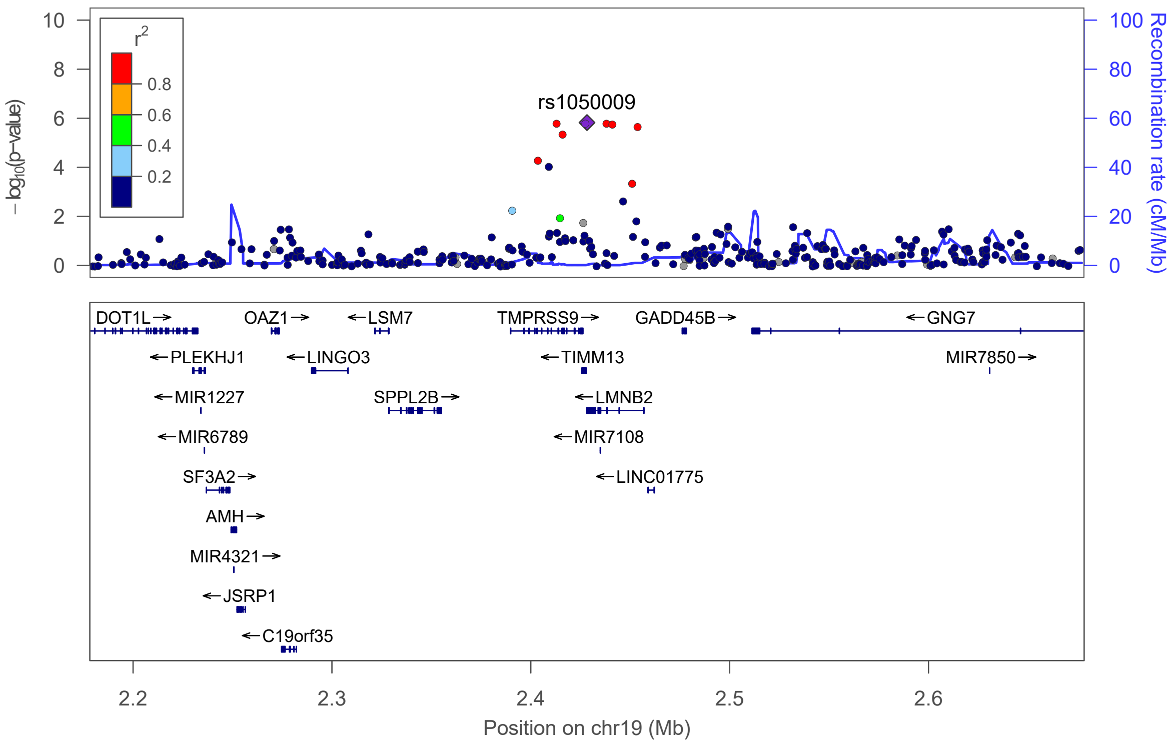

rs1050009
r2
0.8
0.6
0.4
0.2
−log10(p-value)
Recombination rate (cM/Mb)
DOT1L
OAZ1
LSM7
TMPRSS9
GADD45B
GNG7
PLEKHJ1
LINGO3
TIMM13
MIR7850
MIR1227
SPPL2B
LMNB2
MIR6789
MIR7108
SF3A2
LINC01775
AMH
MIR4321
JSRP1
C19orf35
2.2
2.3
2.4
2.5
2.6
Position on chr19 (Mb)

Figure S2

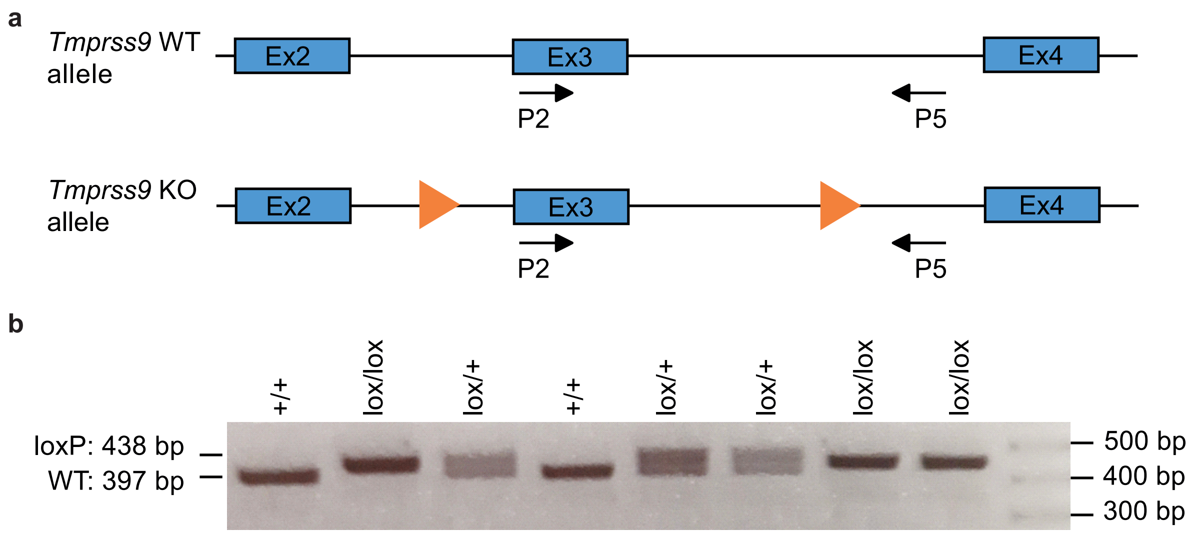
a
Tmprss9 WT allele
Ex2
Ex3
Ex4
P2
P5
Tmprss9 KO allele
Ex2
Ex3
Ex4
P2
P5
b
+/+
lox/lox
lox/+
+/+
lox/+
lox/+
lox/lox
lox/lox
loxP: 438 bp
WT: 397 bp
500 bp
400 bp
300 bp

Figure S3

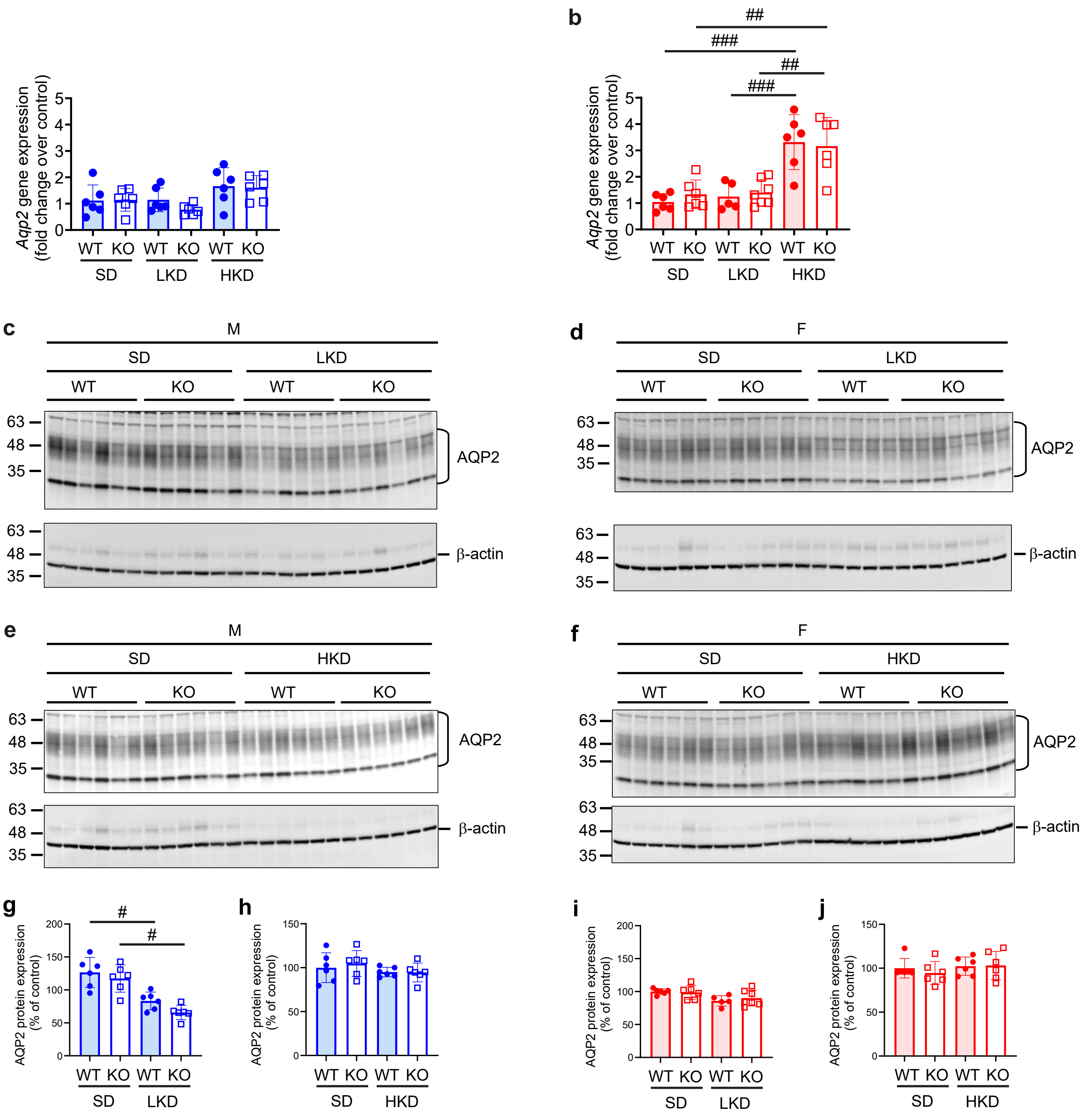
a
Aqp2 gene expression (fold change over control)
WT KO WT KO WT KO
SD LKD HKD
b
##
###
##
###
c
M
SD LKD
WT KO WT KO
63
48
35
AQP2
β-actin
d
F
e
HKD
f
g
#
#
AQP2 protein expression (% of control)
h
i
j

Uncropped Western blots part 1

Figure 4a
Grouped representative Western blot analysis of NCC and beta-actin in kidneys from (a) male WT and KO mice

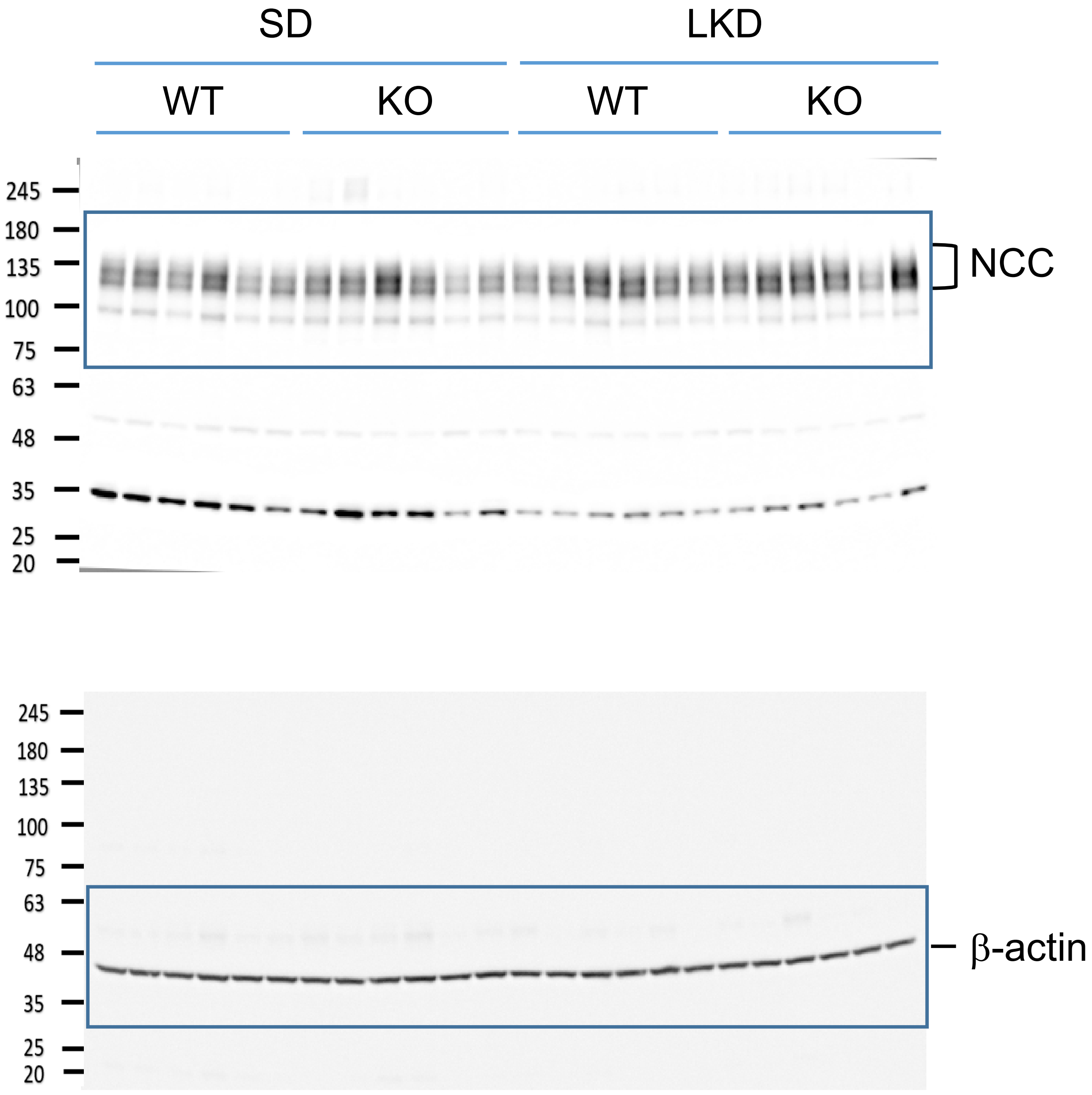

Uncropped Western blots part 1

Figure 4a
Grouped representative Western blot analysis of p53-NCC and beta-actin in kidneys from (a) male WT and KO mice

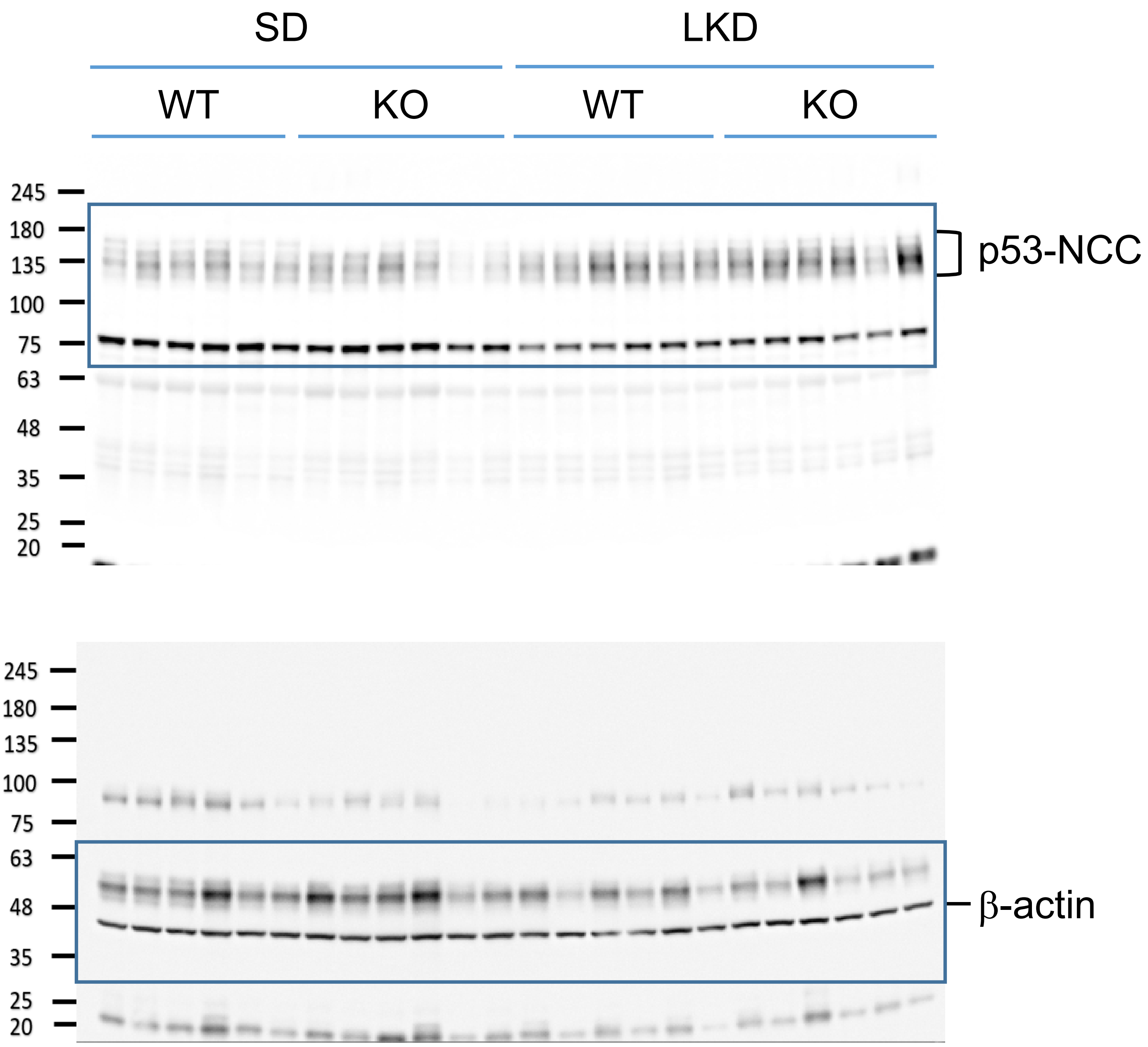

Uncropped Western blots part 1

Figure 4a

Grouped representative Western blot analysis of p58-NCC and beta-actin in kidneys from (a) male WT and KO mice

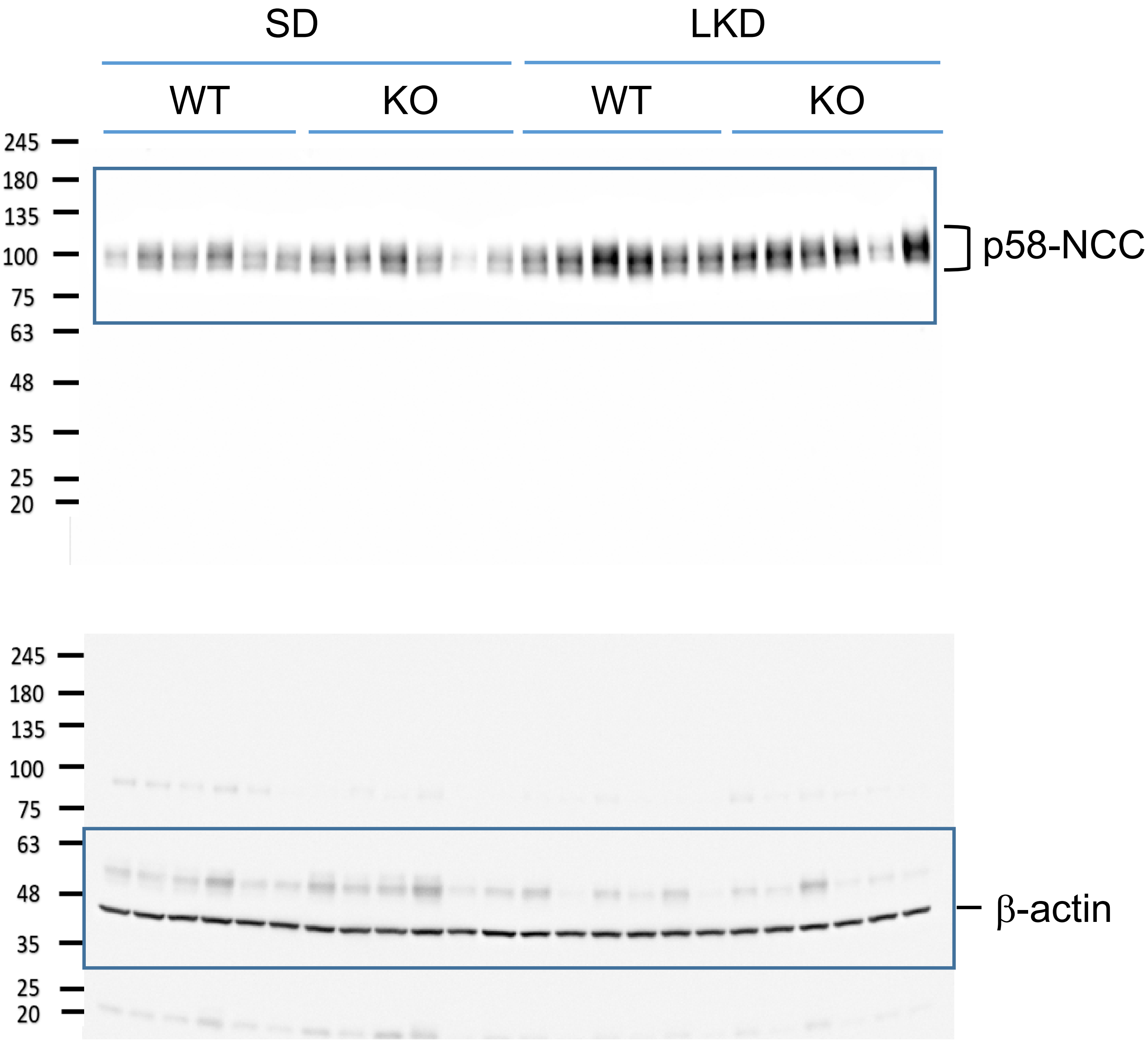

Uncropped Western blots part 1

Figure 4b

Grouped representative Western blot analysis of NCC and beta-actin in kidneys from (b) female WT and KO mice

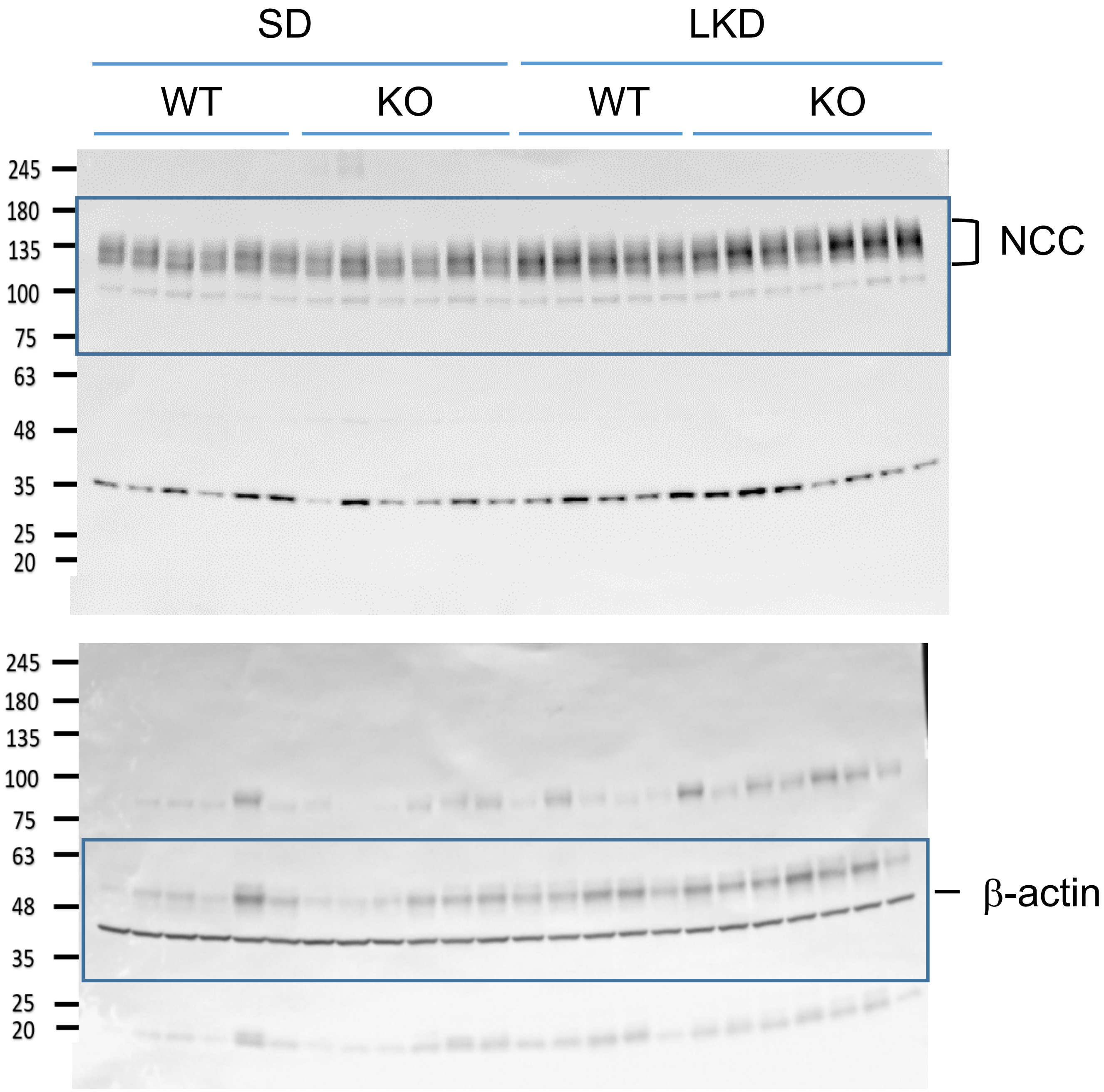

Uncropped Western blots part 1

Figure 4b

Grouped representative Western blot analysis of p53-NCC and beta-actin in kidneys from (b) female WT and KO mice

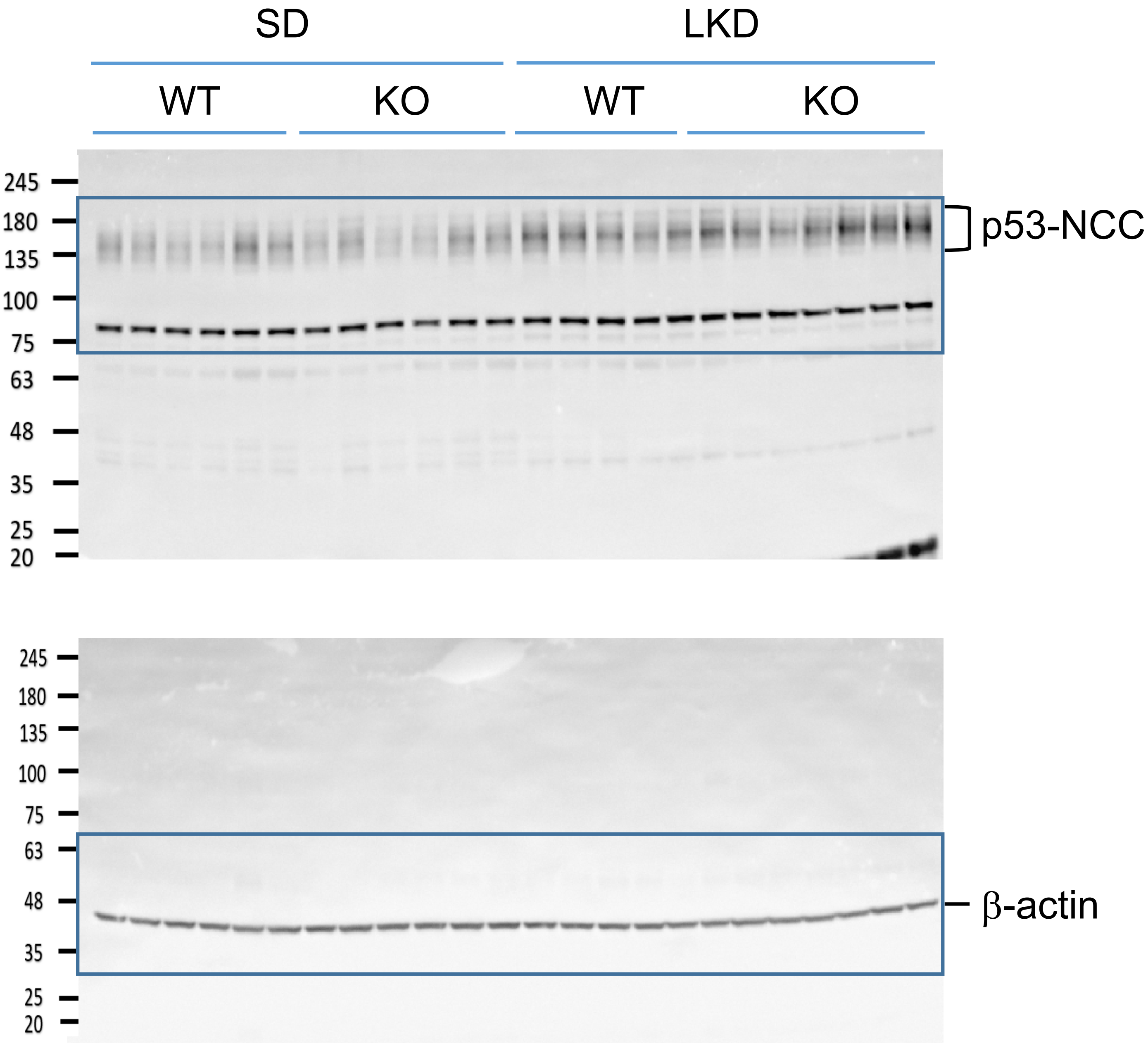

Uncropped Western blots part 1

Figure 4b
Grouped representative Western blot analysis of p58-NCC and beta-actin in kidneys from (b) female WT and KO mice

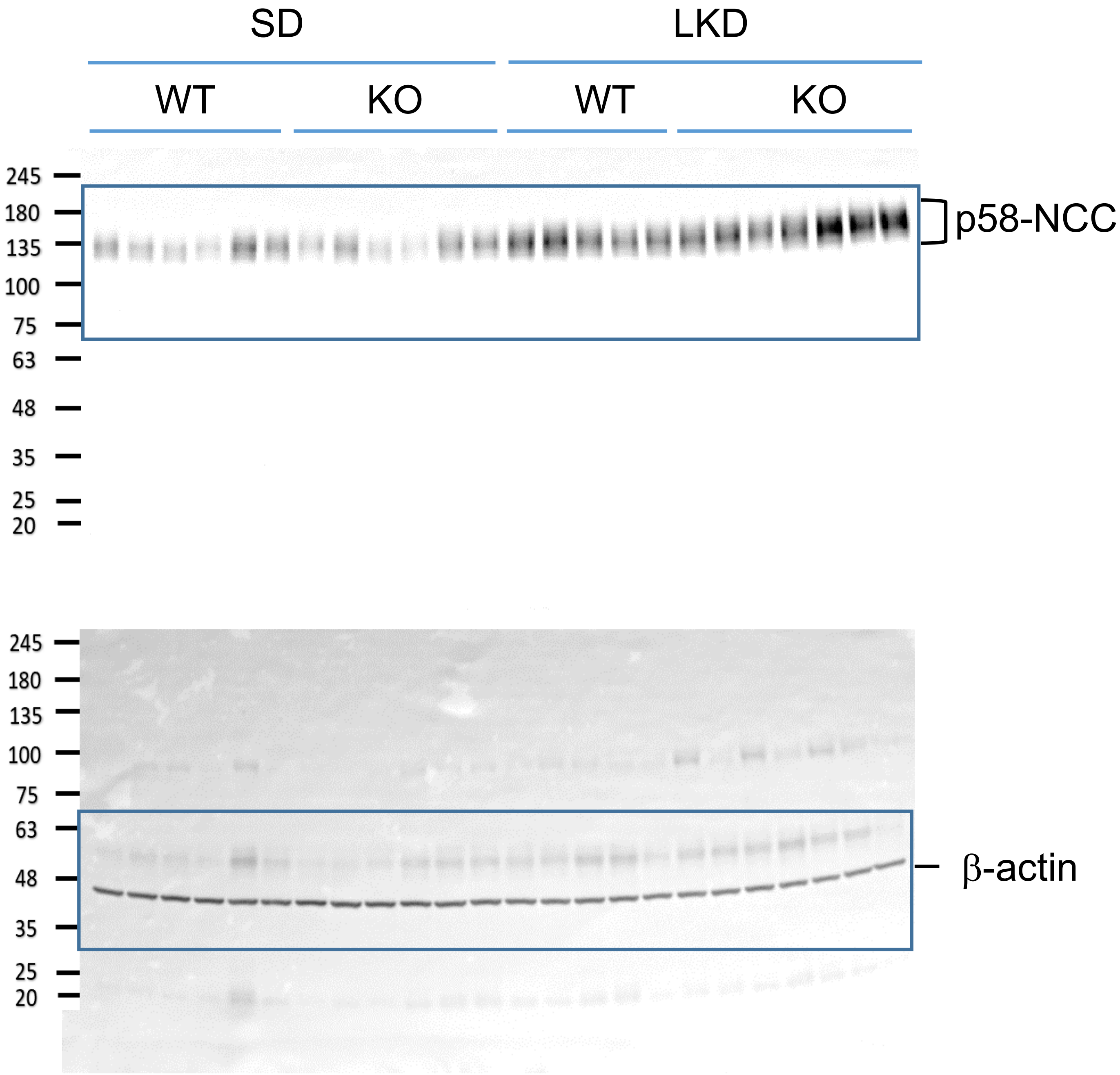

Uncropped Western blots part 2

Figure 5a

Grouped representative Western blot analysis of NCC and beta-actin in kidney of (a) male WT and KO mice

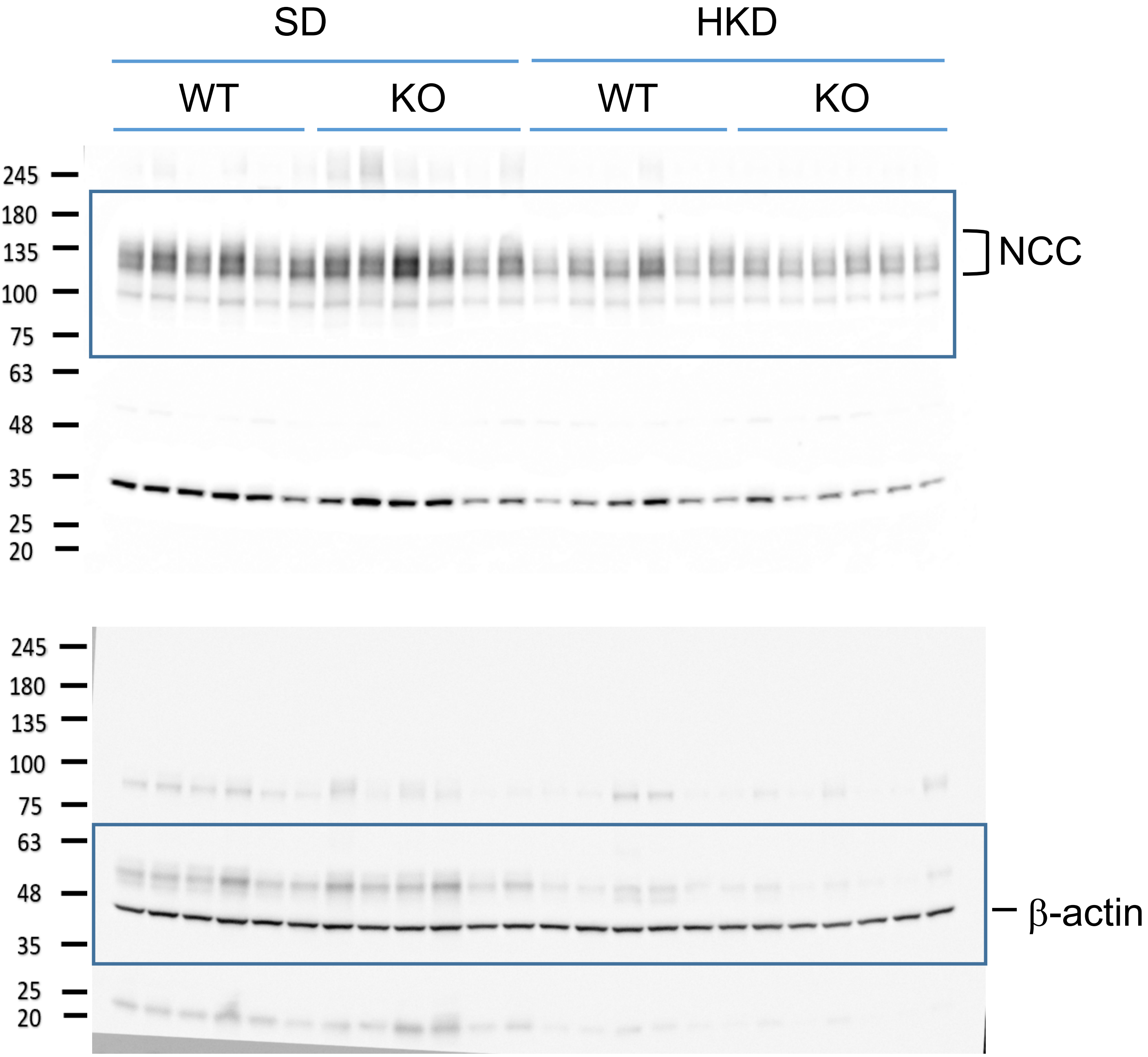

Uncropped Western blots part 2

Figure 5a

Grouped representative Western blot analysis of p53-NCC and beta-actin in kidney of (a) male WT and KO mice

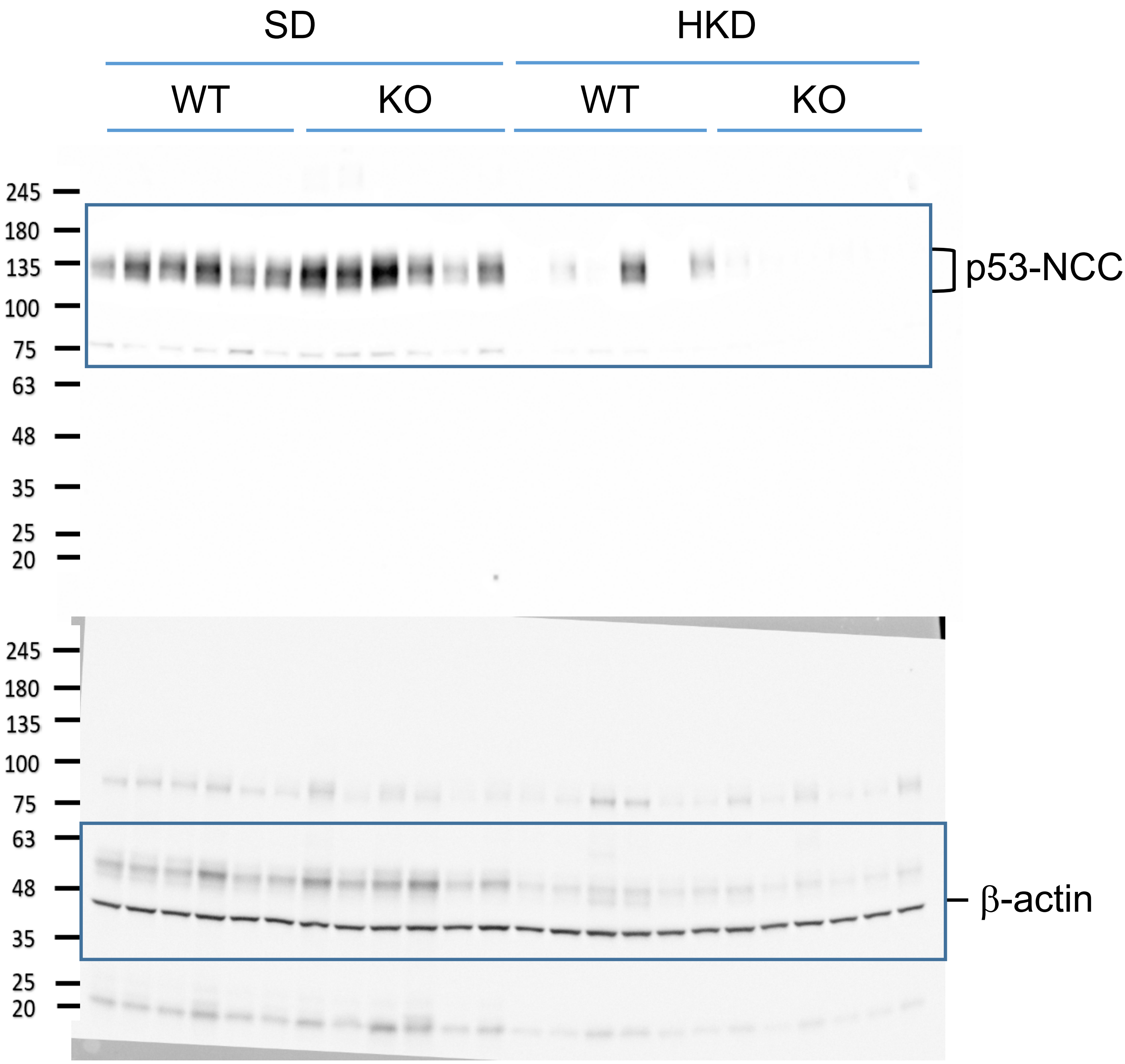

Uncropped Western blots part 2

Figure 5a
Grouped representative Western blot analysis of p58-NCC and beta-actin in kidney of (a) male WT and KO mice

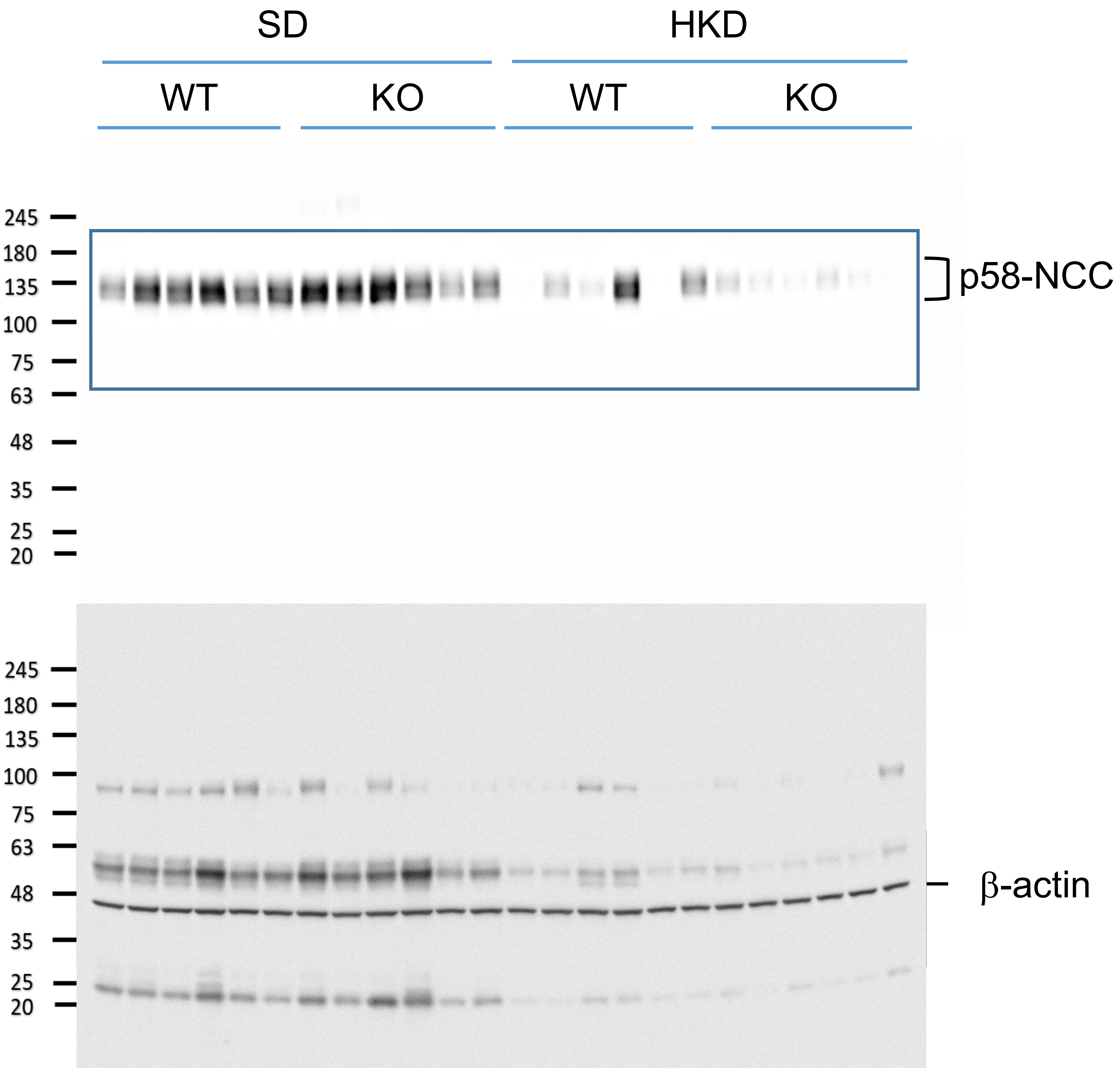

Uncropped Western blots part 2

Figure 5b
Grouped representative Western blot analysis of NCC and beta-actin in kidney of (b) female WT and KO mice

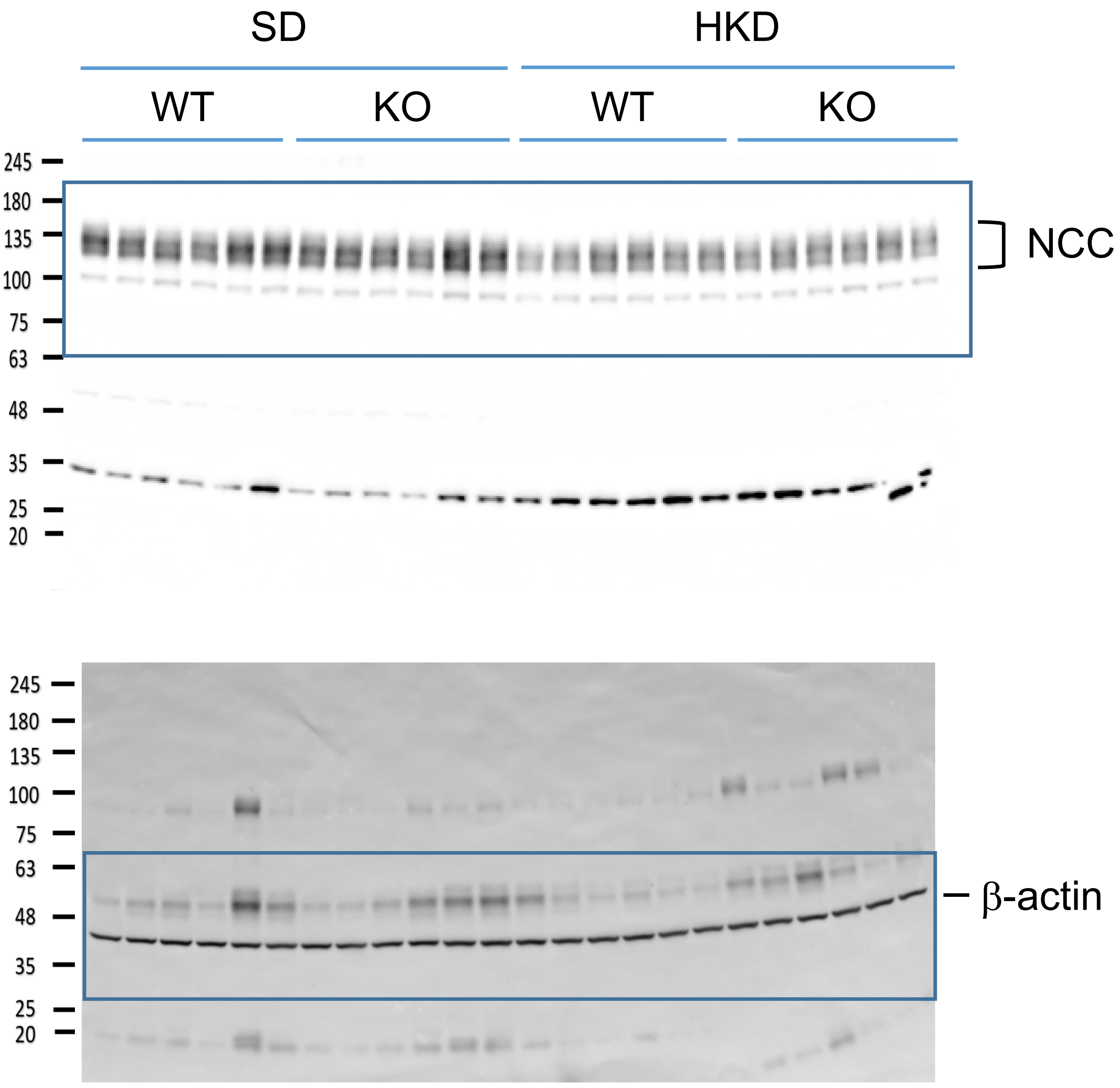

Uncropped Western blots part 2

Figure 5b

Grouped representative Western blot analysis of p53-NCC and beta-actin in kidney of (b) female WT and KO mice

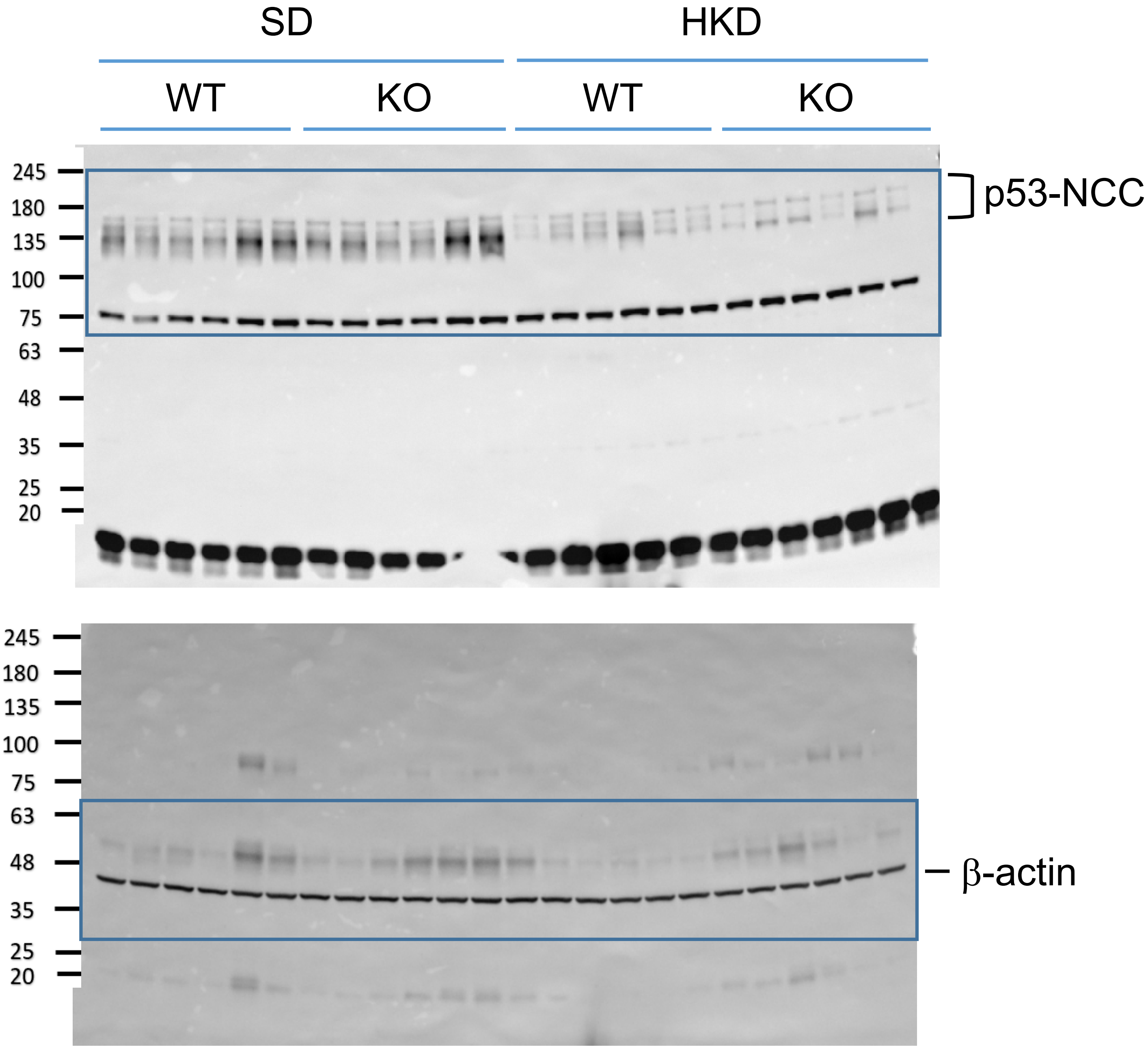

Uncropped Western blots part 2

Figure 5b
Grouped representative Western blot analysis of p58-NCC and beta-actin in kidney of (b) female WT and KO mice

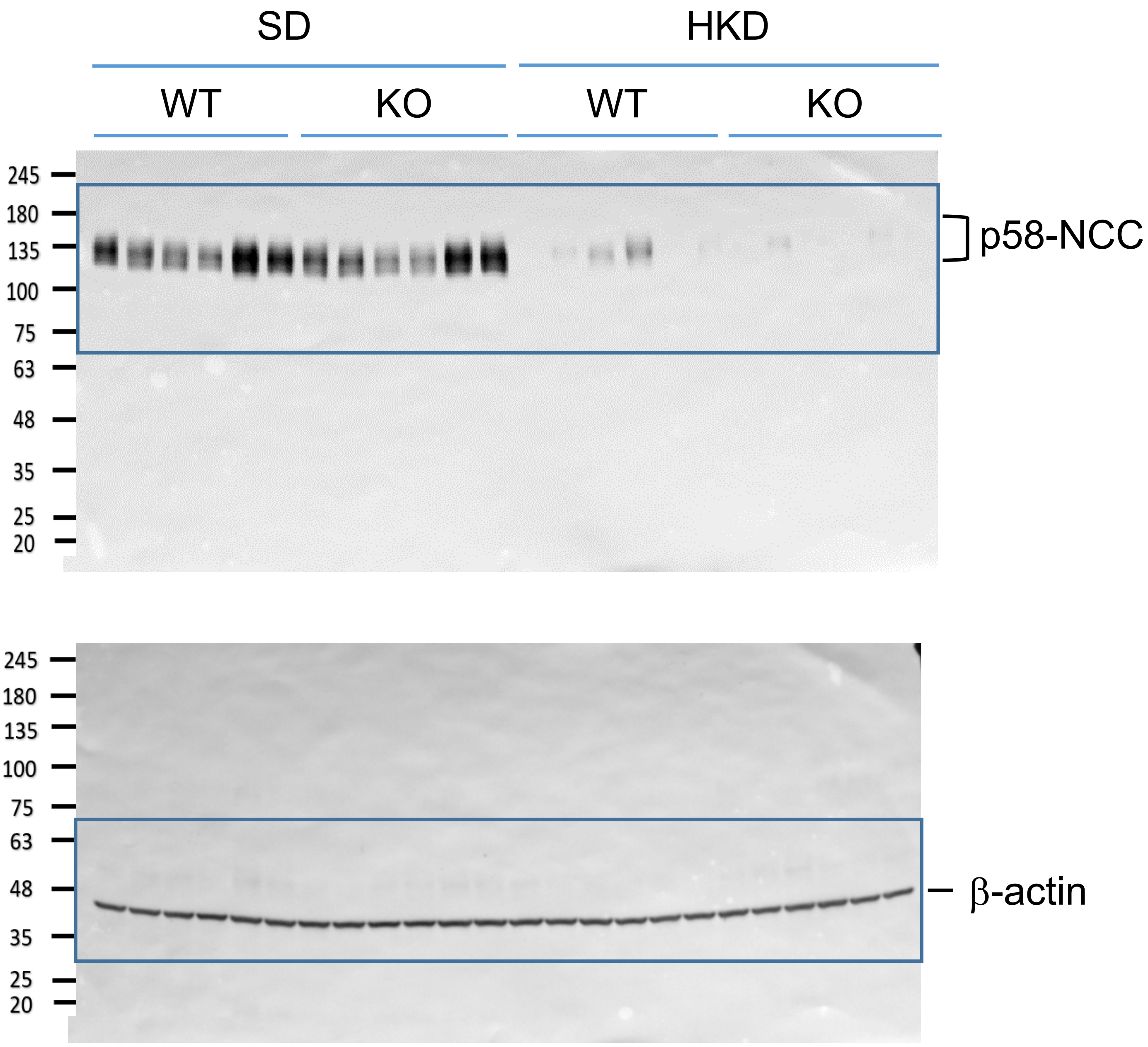

Uncropped Western blots part 3

Figure 6a Grouped representative Western blot analysis of alpha ENaC and beta-actin in kidney of (a) male WT and KO mice

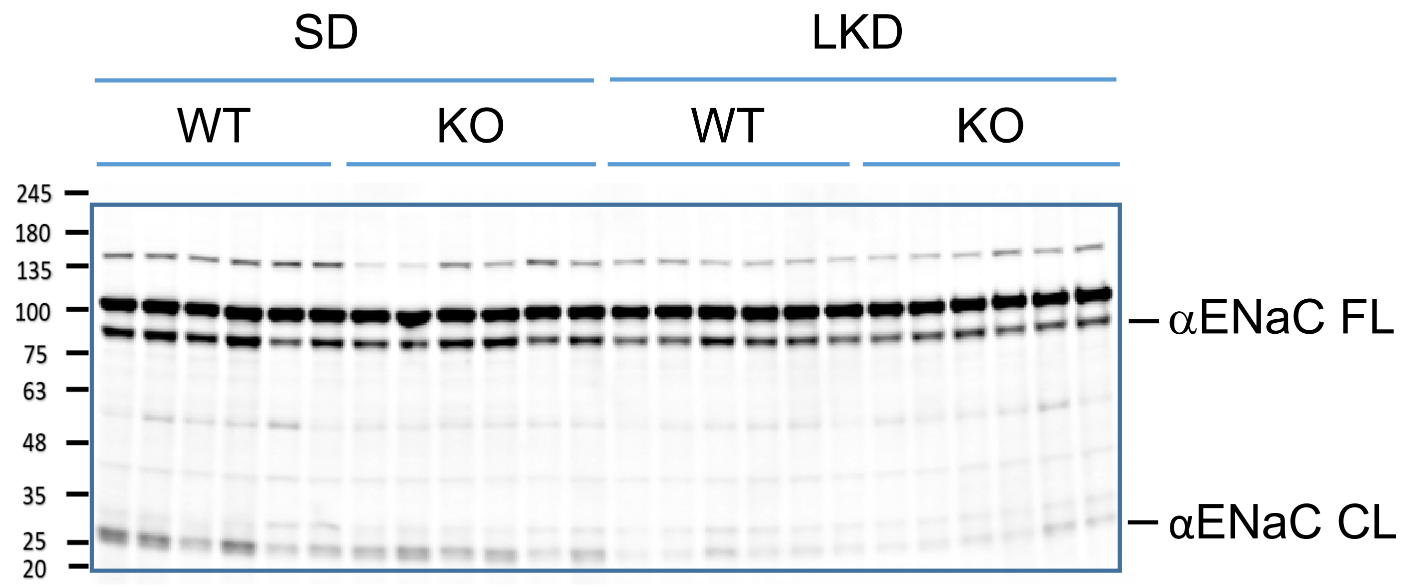

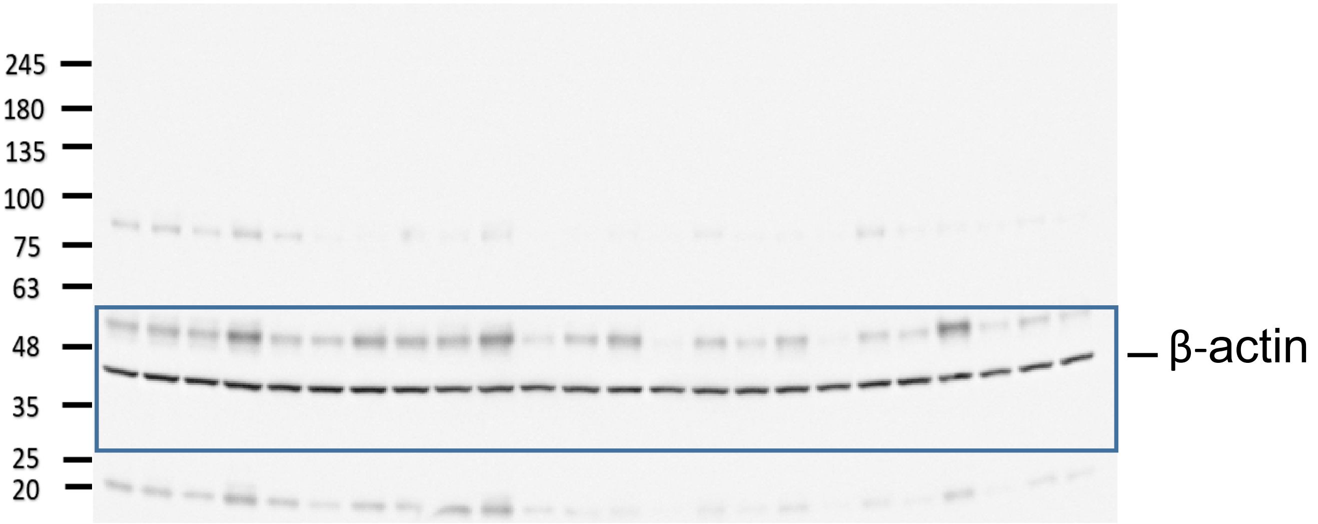

Uncropped Western blots part 3

Figure 6a Grouped representative Western blot analysis of gamma ENaC and beta-actin in kidney of (a) male WT and KO mice

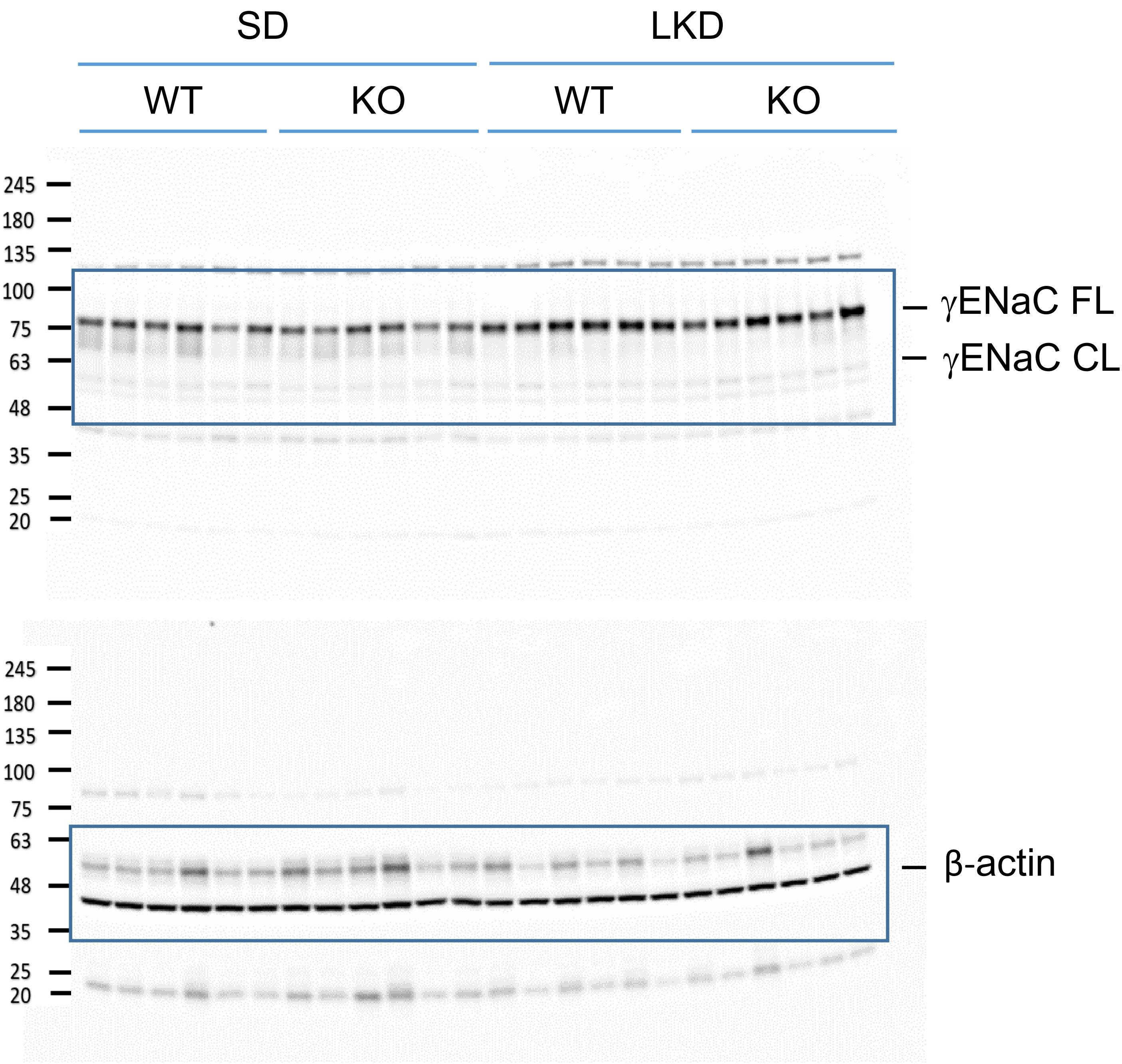

Uncropped Western blots part 3

Figure 6b Grouped representative Western blot analysis of alpha ENaC in kidney of (b) female WT and KO mice

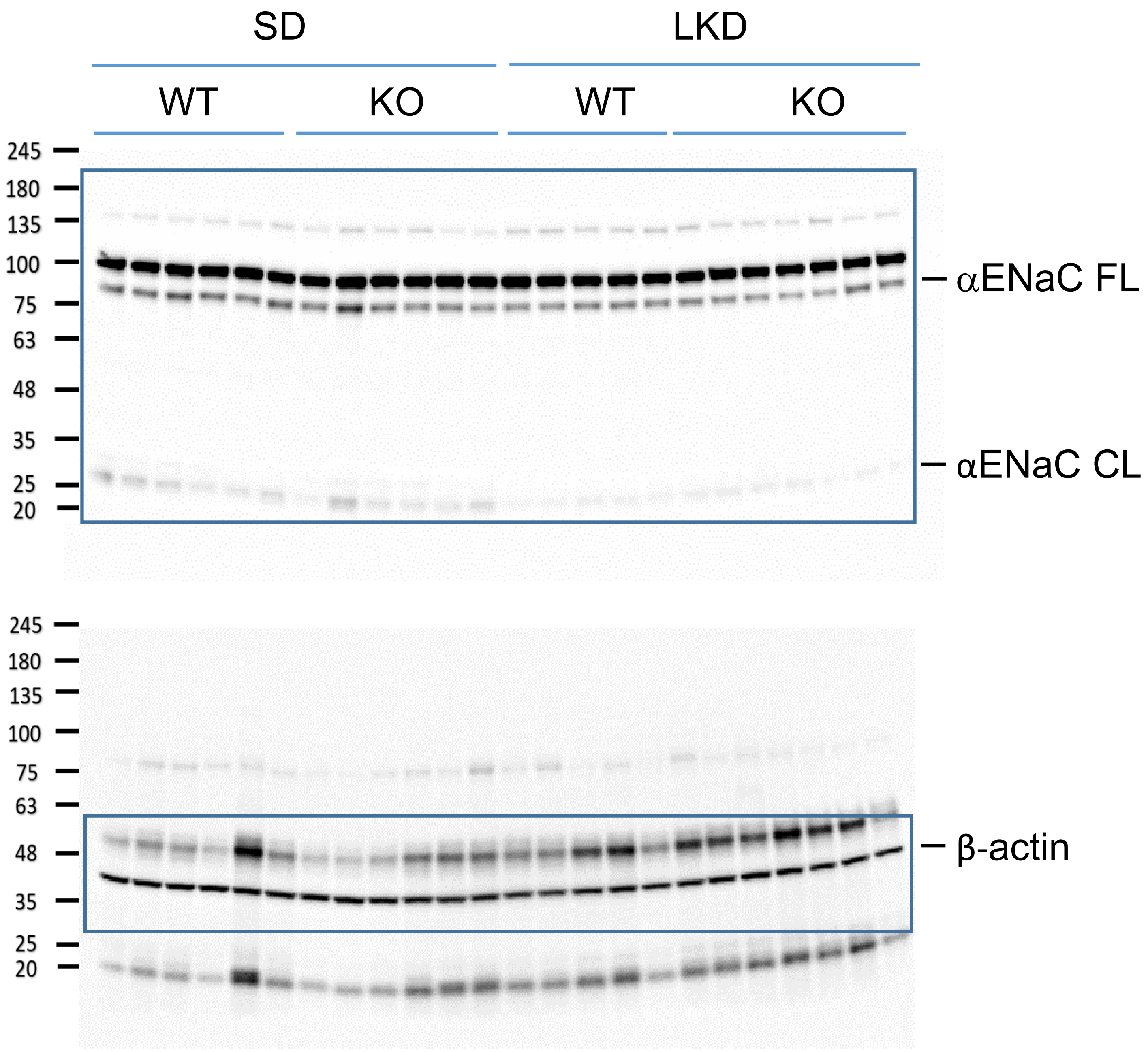

Uncropped Western blots part 3

Figure 6b Grouped representative Western blot analysis of gamma ENaC and beta-actin in kidney of (b) female WT and KO mice

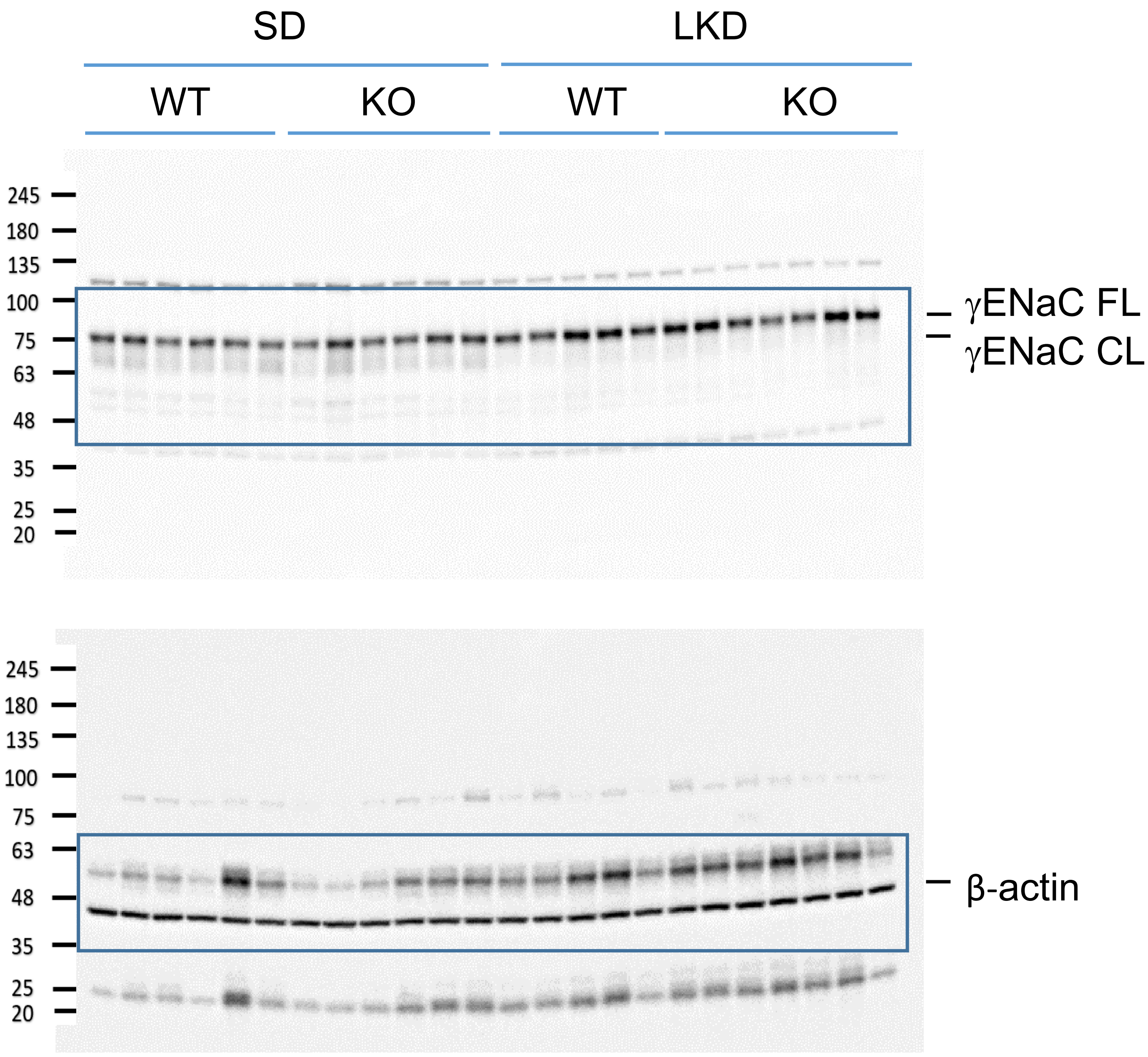

Uncropped Western blots part 4

Figure 7a Grouped representative Western blot analysis of alpha ENaC and beta-actin in kidney of (a) male WT and KO mice

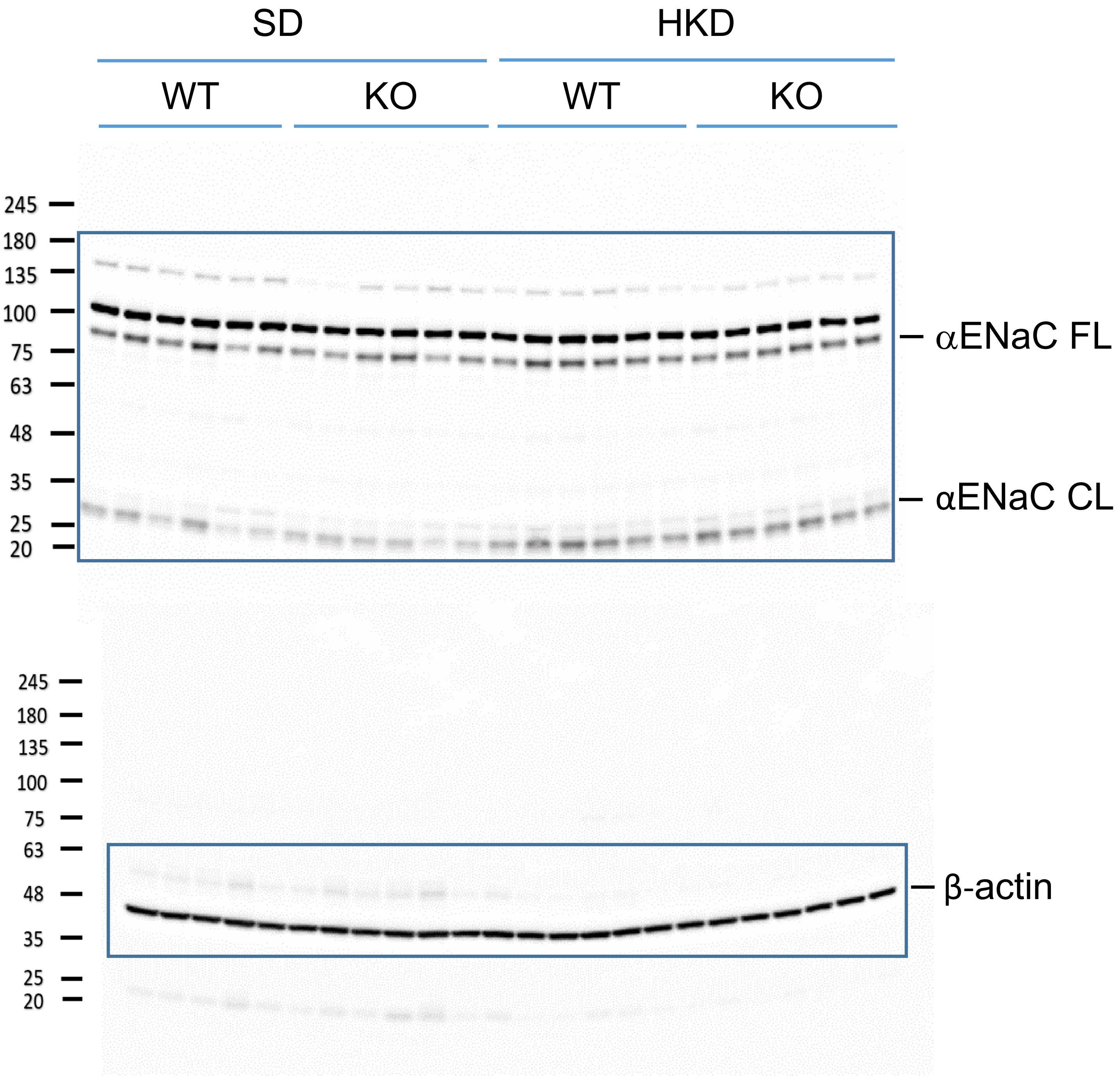

Uncropped Western blots part 4

Figure 7a Grouped representative Western blot analysis of gamma ENaC and beta-actin in kidney of (a) male WT and KO mice

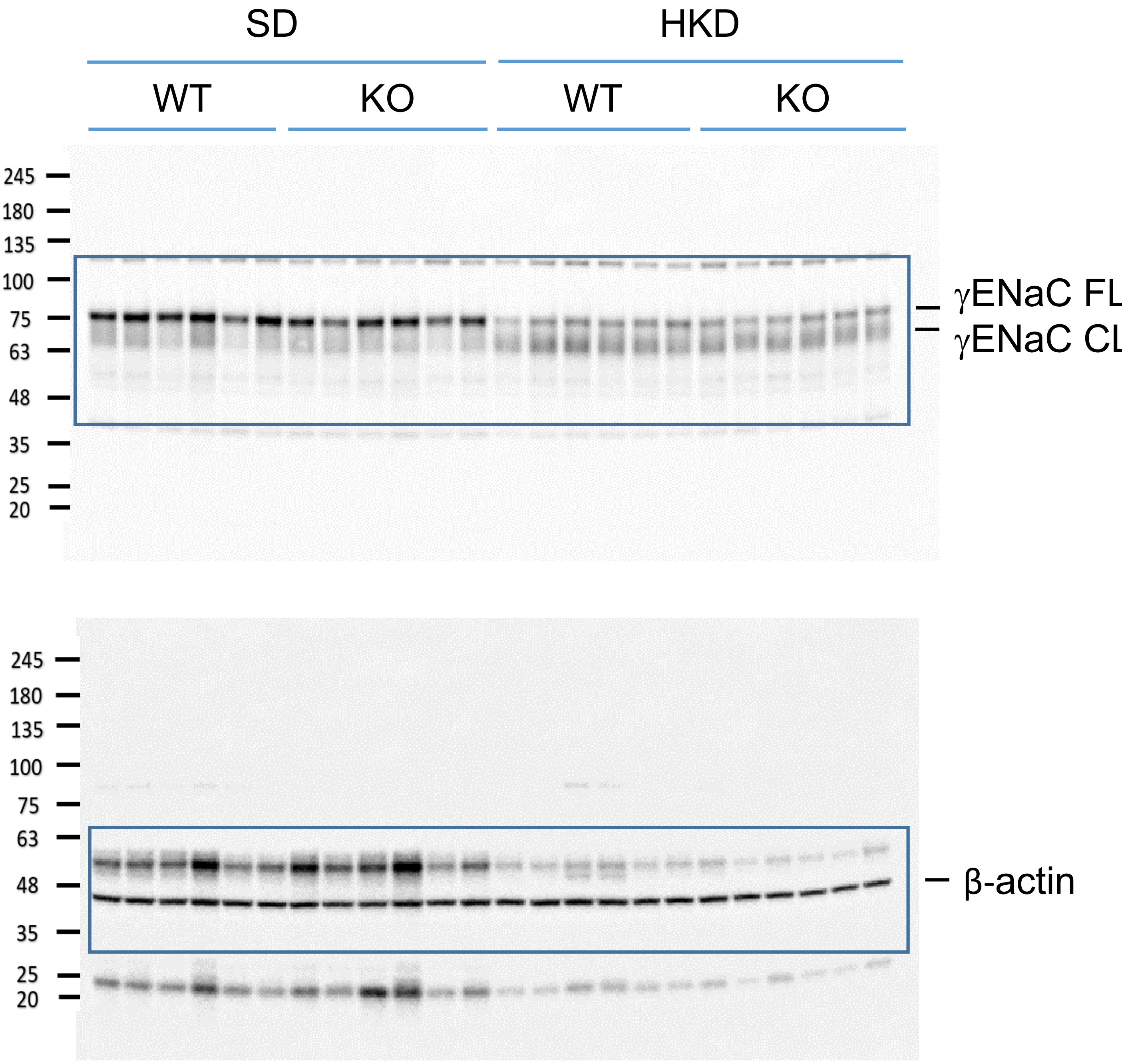

Uncropped Western blots part 4

Figure 7b Grouped representative Western blot analysis of alpha ENaC and beta-actin in kidney of (b) female WT and KO mice

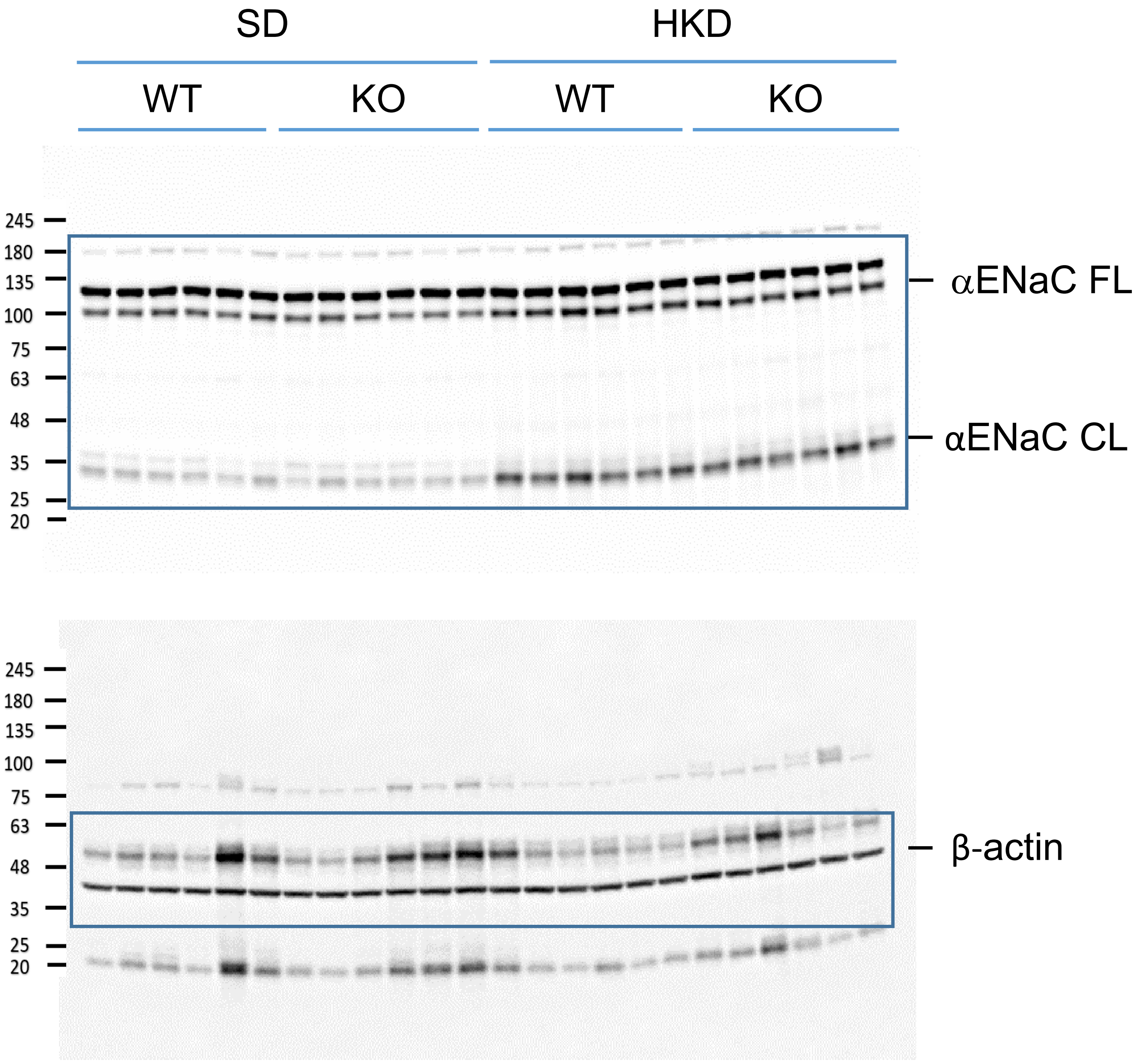

Uncropped Western blots part 4

Figure 7b Grouped representative Western blot analysis of gamma ENaC and beta-actin in kidney of (b) female WT and KO mice

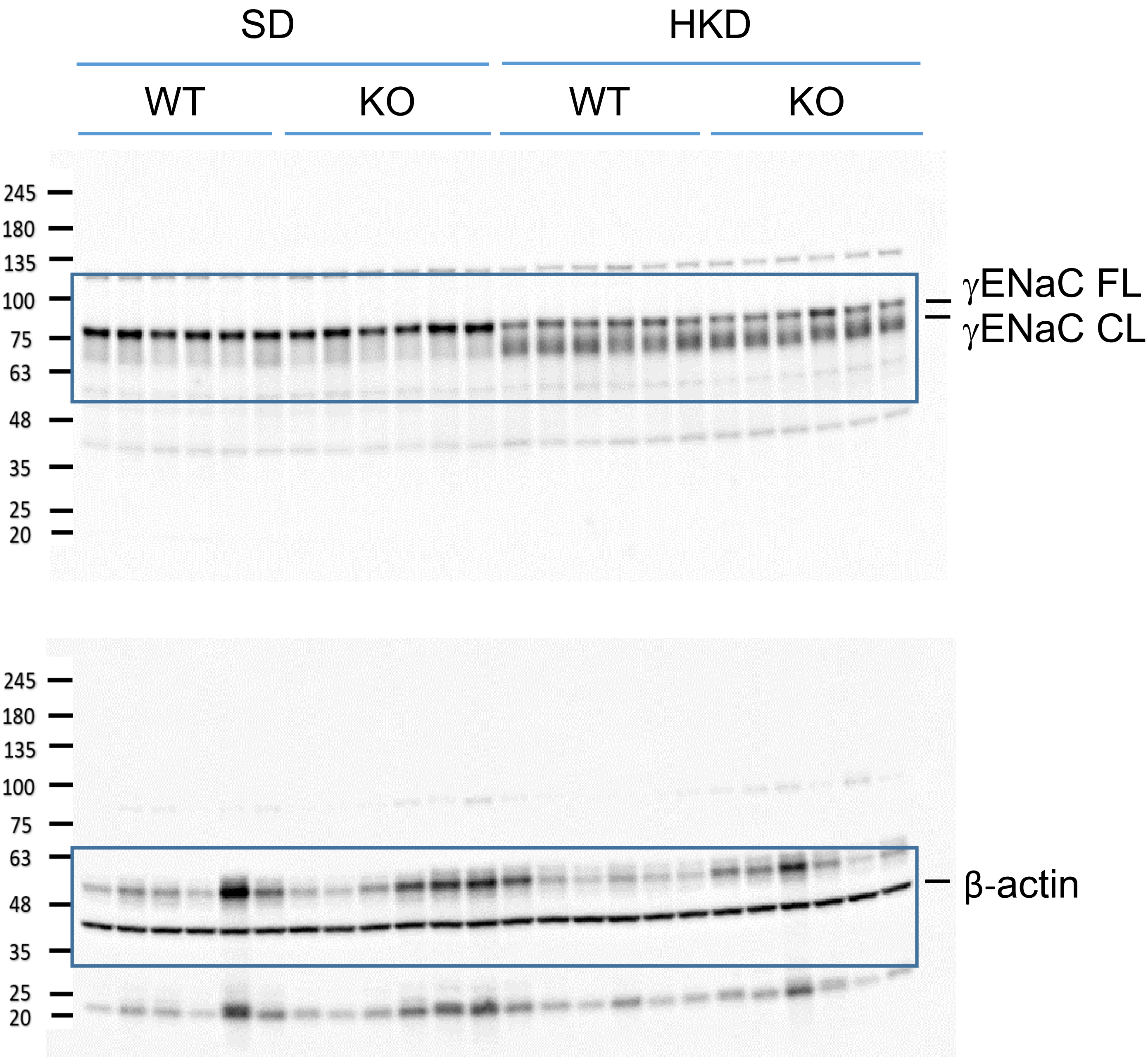

Uncropped Western blots Part 5

Figure 8c
Grouped representative Western blot analysis of NHE3 and beta-actin in kidneys from (c) male WT and KO mice

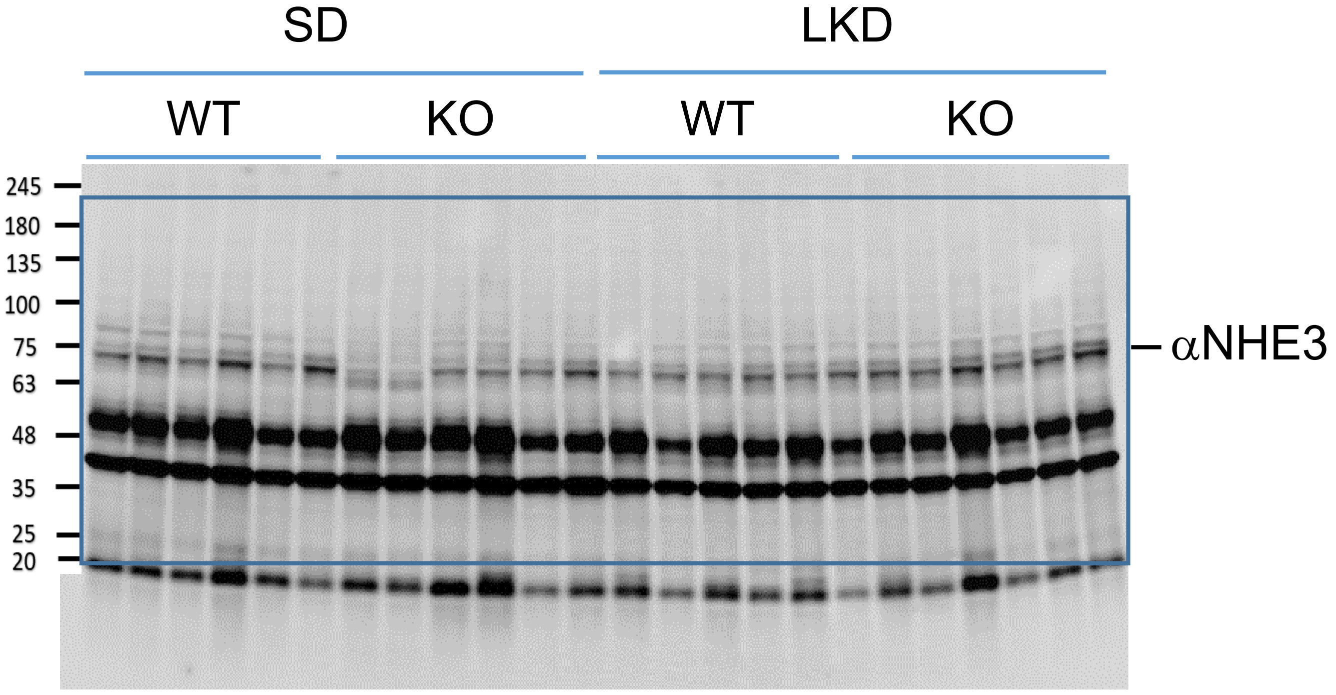

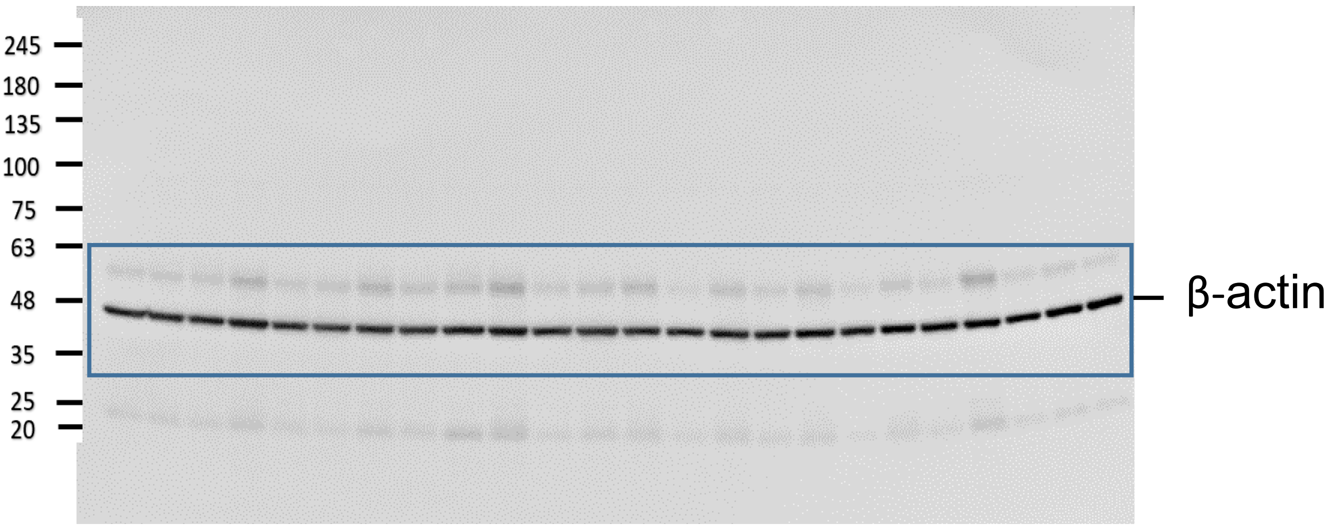

Uncropped Western blots Part 5

Figure 8d
Grouped representative Western blot analysis of NHE3 and beta-actin in kidneys from (d) female WT and KO mice

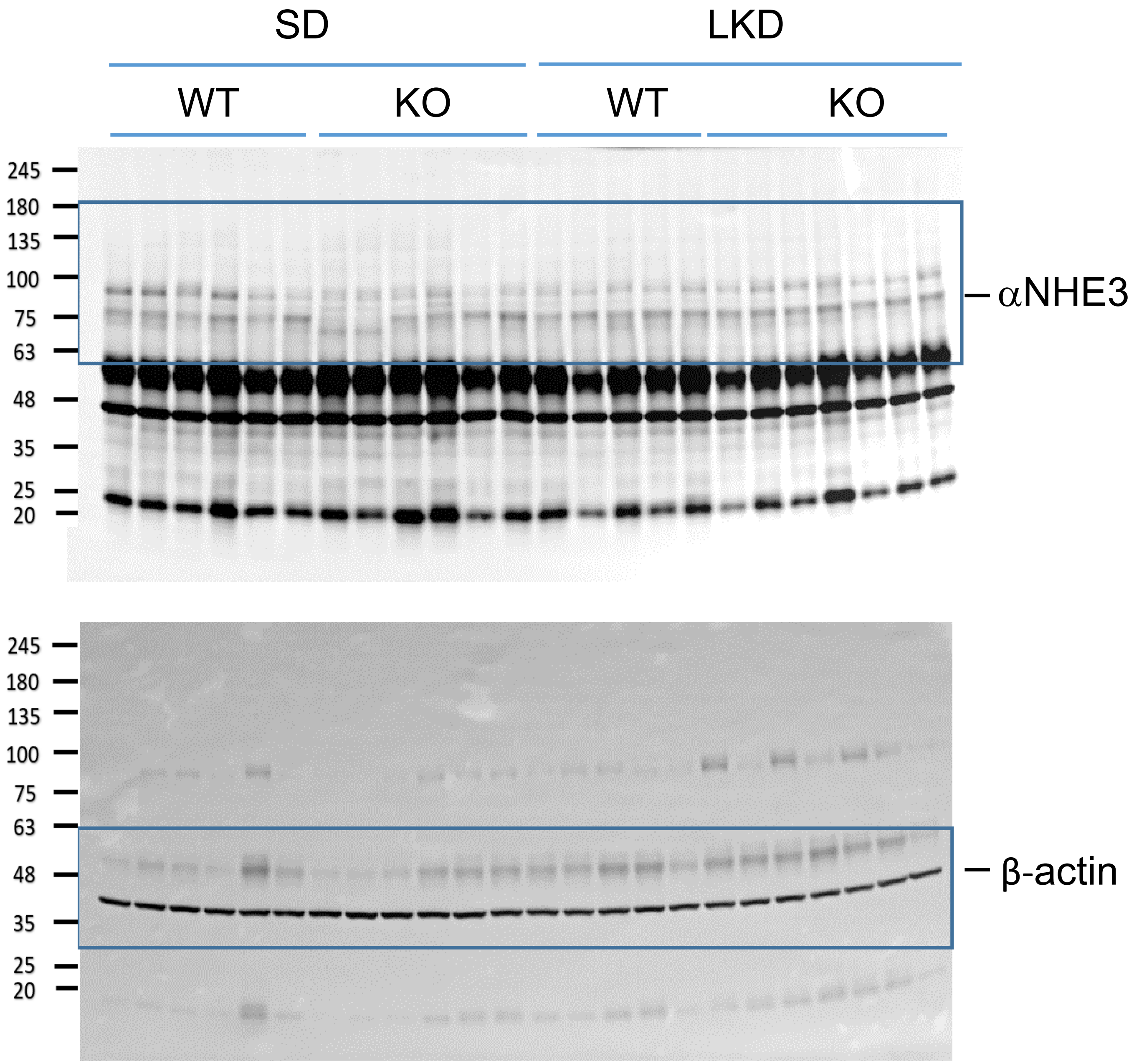

Uncropped Western blots Part 5

Figure 8e
Grouped representative Western blot analysis of NHE3 and beta-actin in kidneys from (e) male WT and KO mice

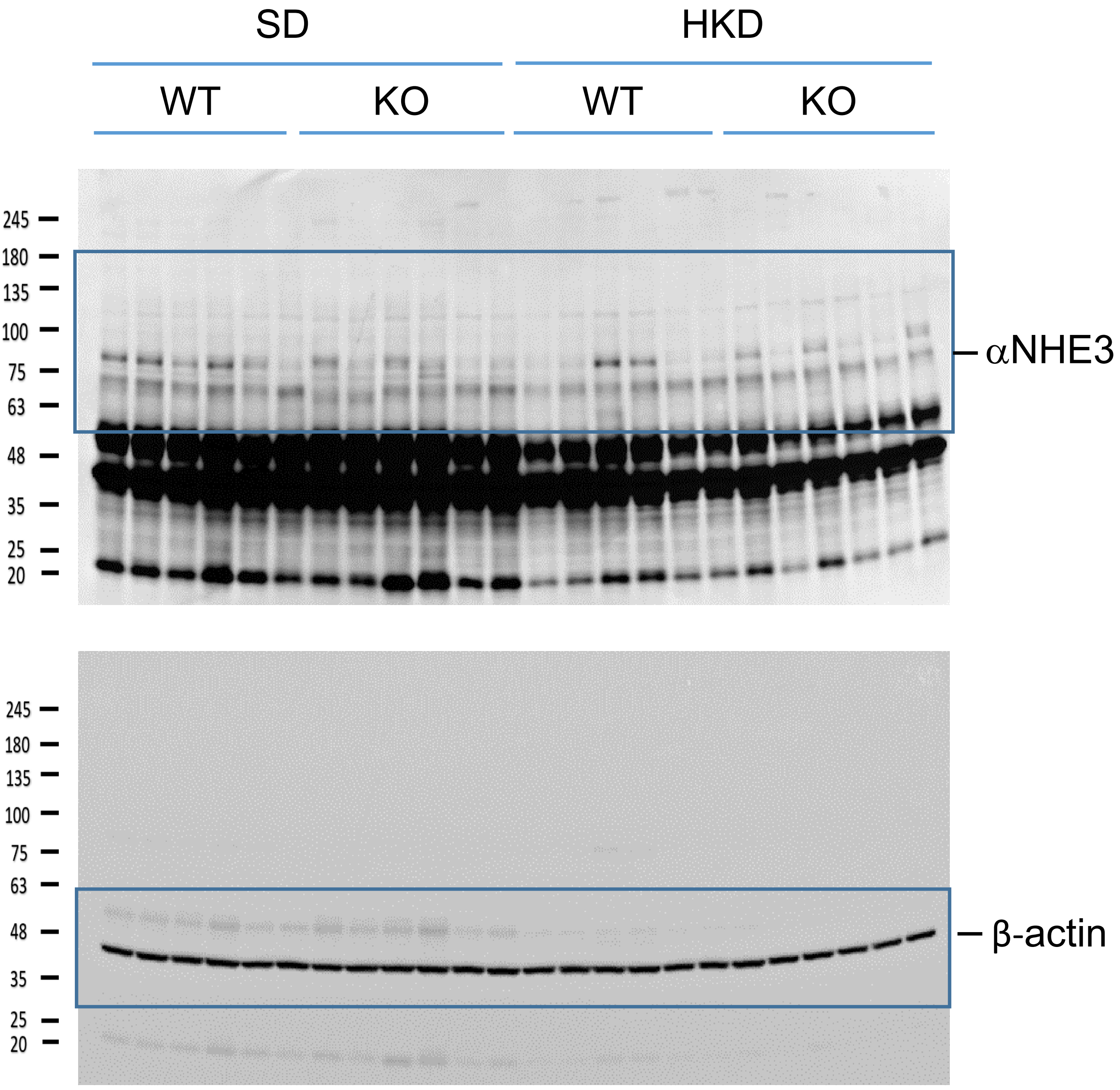

Uncropped Western blots Part 5

Figure 8f
Grouped representative Western blot analysis of NHE3 and beta-actin in kidneys from (f) female WT and KO mice

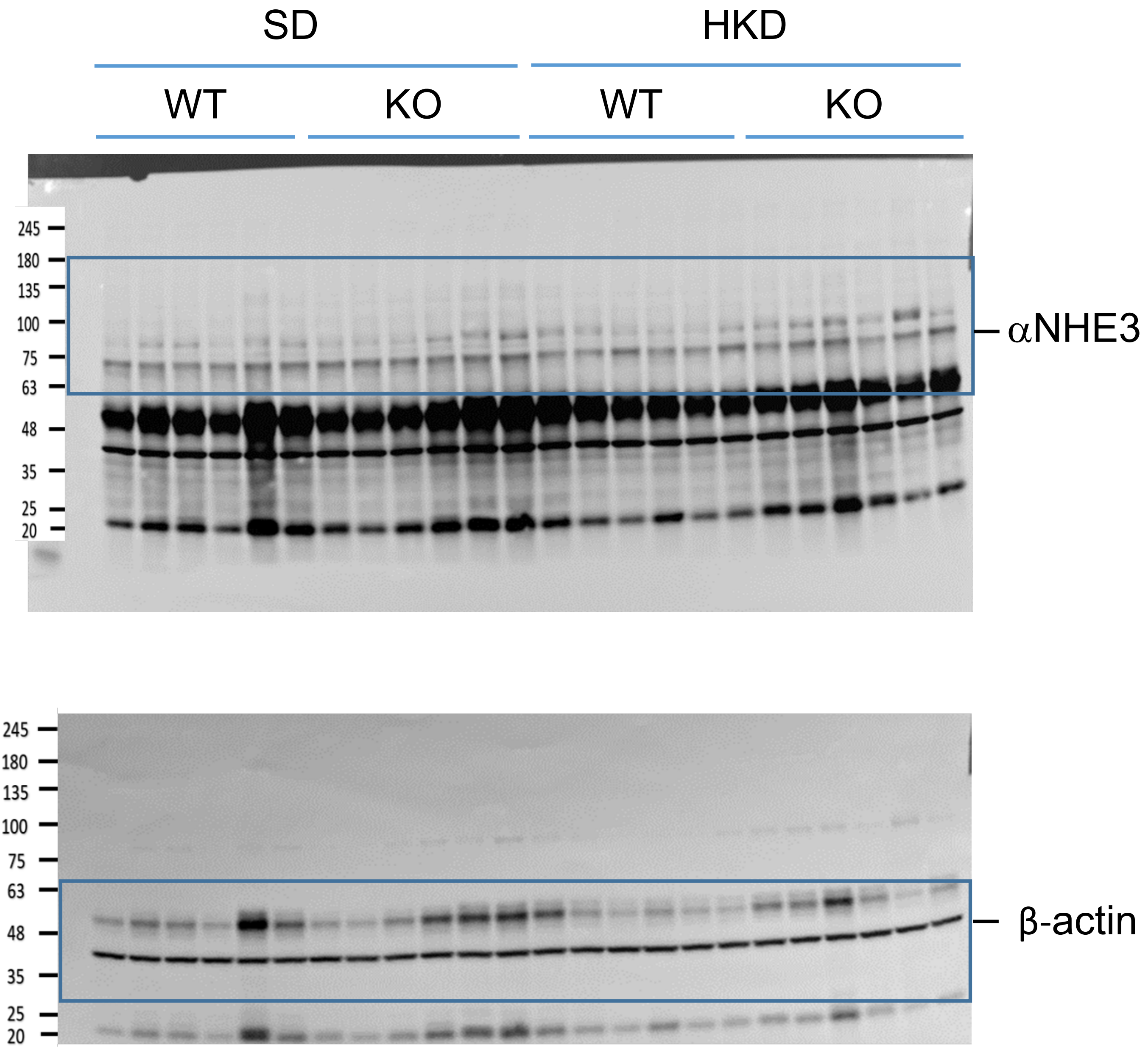

Uncropped Western blots Part 6

Figure S3c Grouped representative Western blot analysis of AQP2 and beta-actin in kidneys from (c) male WT and KO mice

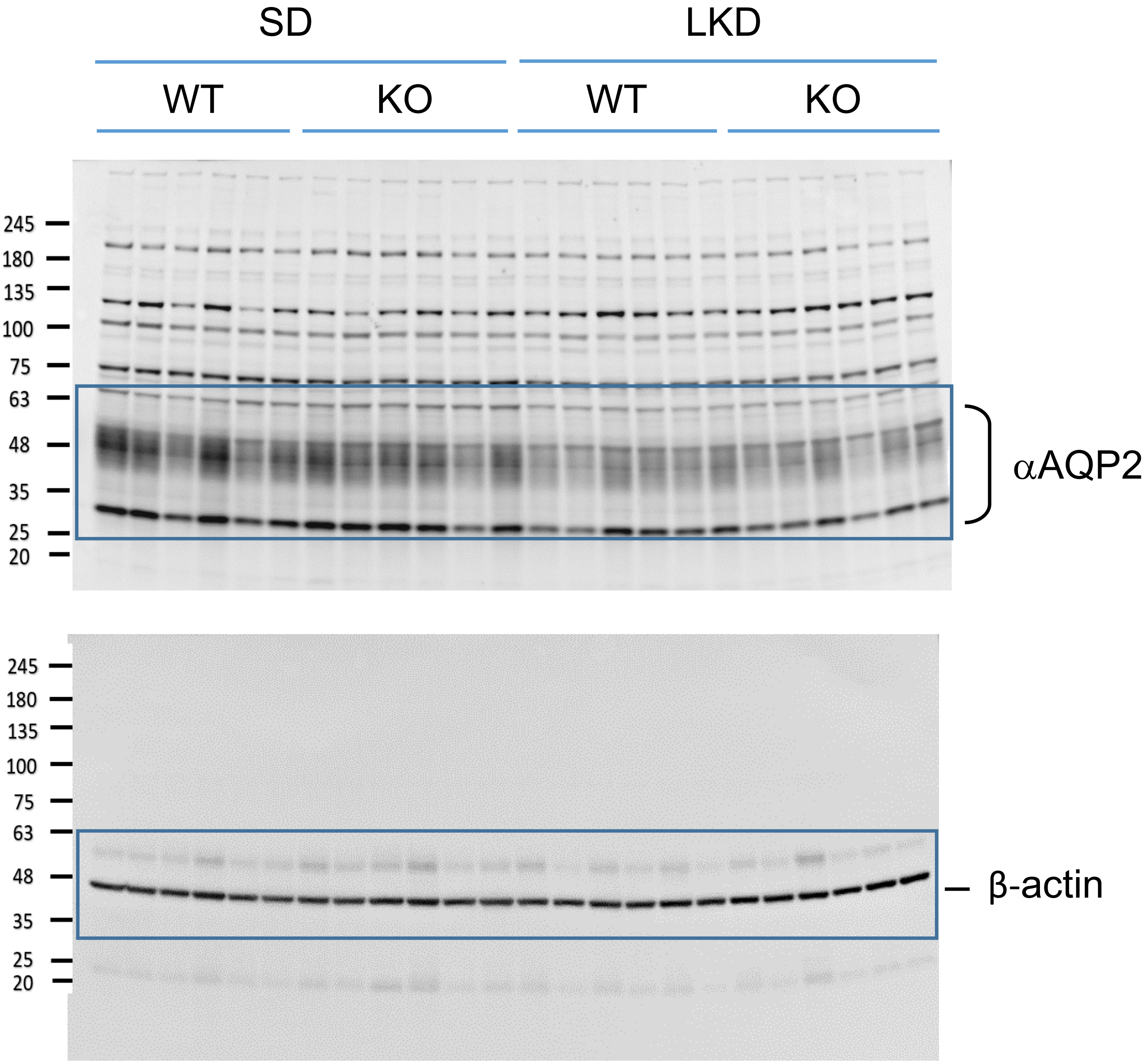

Uncropped Western blots Part 6

Figure S3d Grouped representative Western blot analysis of AQP2 and beta-actin in kidneys from (d) female WT and KO mice

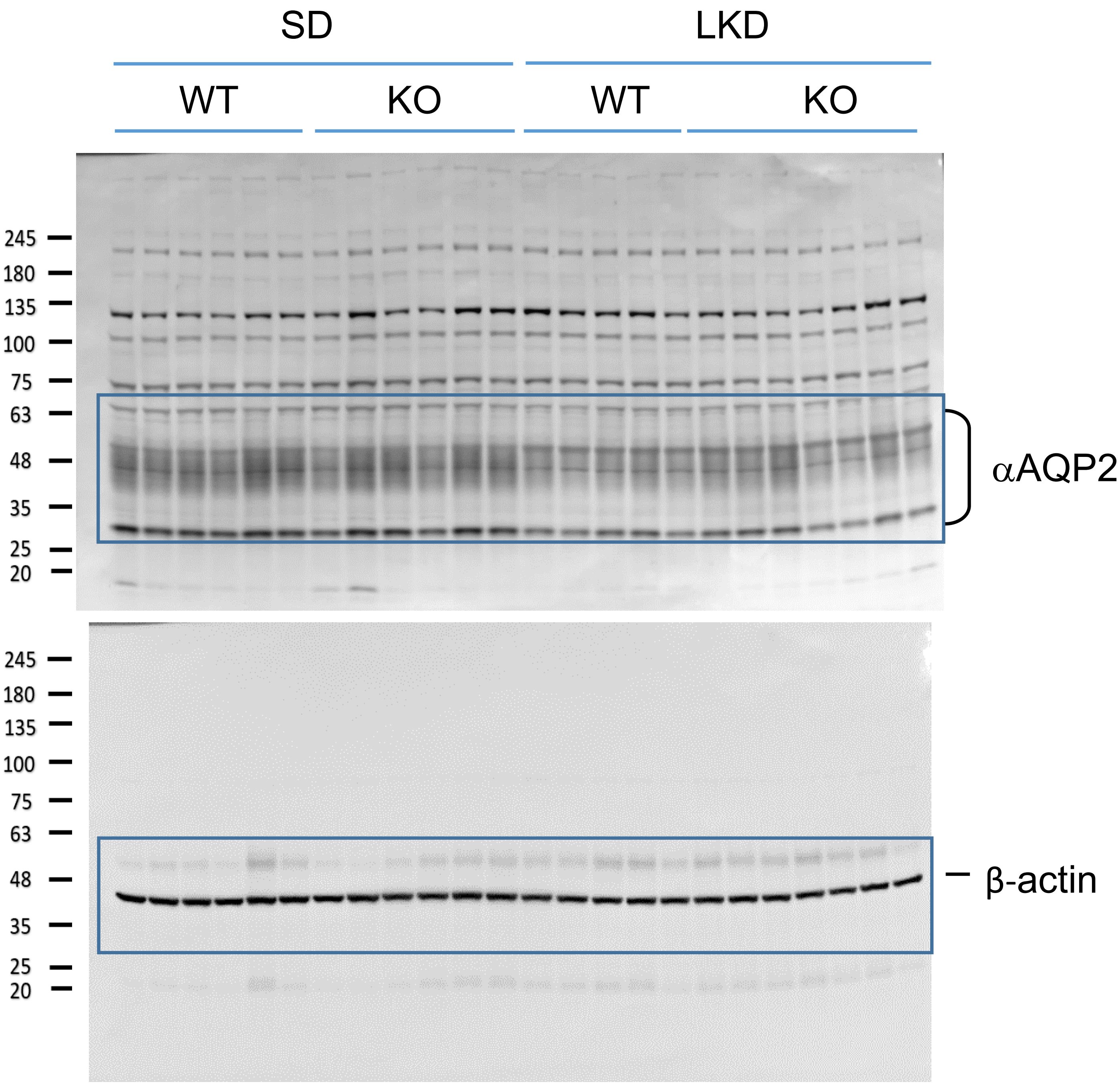

Uncropped Western blots Part 6

Figure S3e Grouped representative Western blot analysis of AQP2 and beta-actin in kidneys from (e) male WT and KO mice

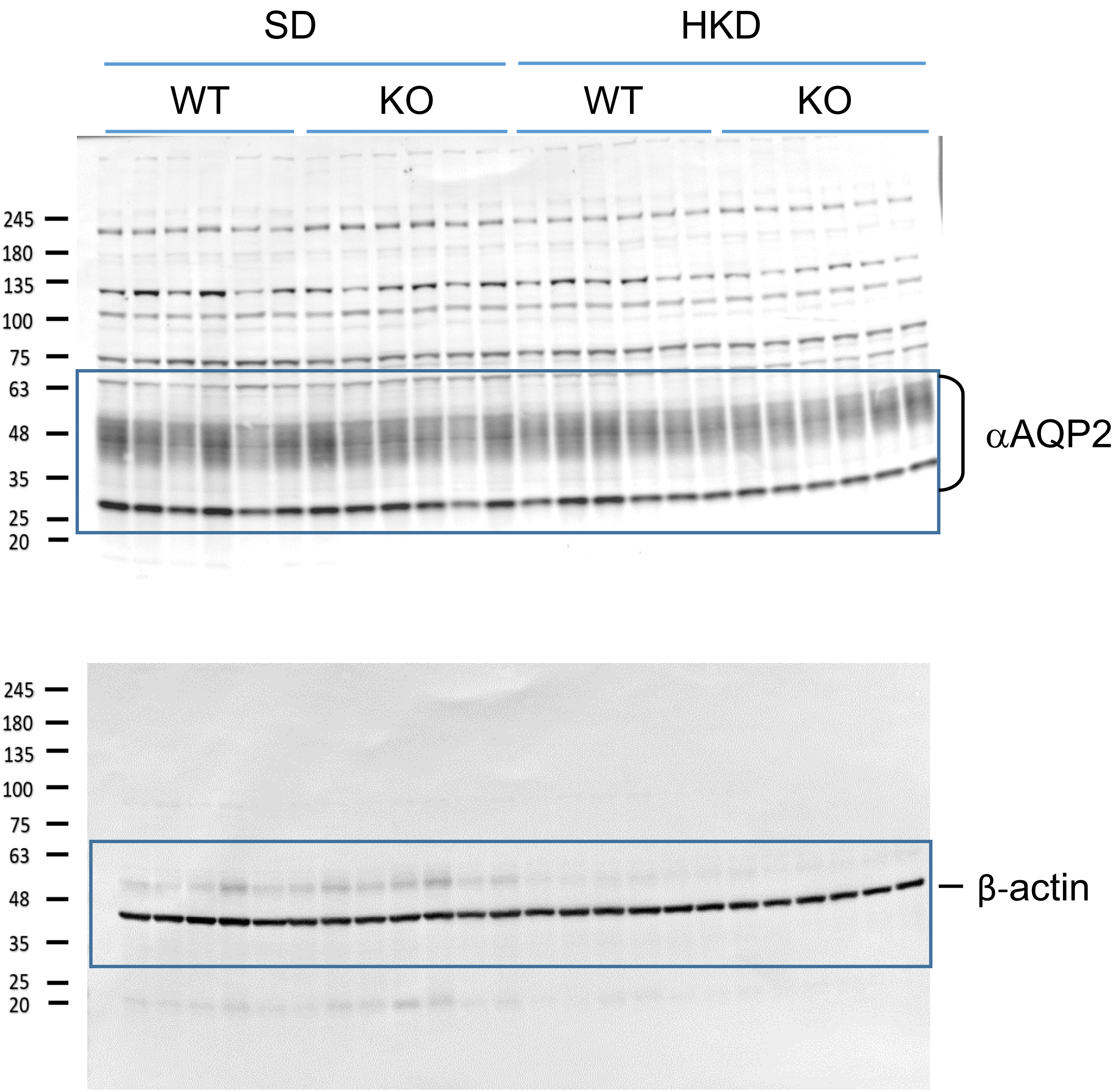

Uncropped Western blots Part 6

Figure S3f Grouped representative Western blot analysis of AQP2 and beta-actin in kidneys from (f) female WT and KO mice

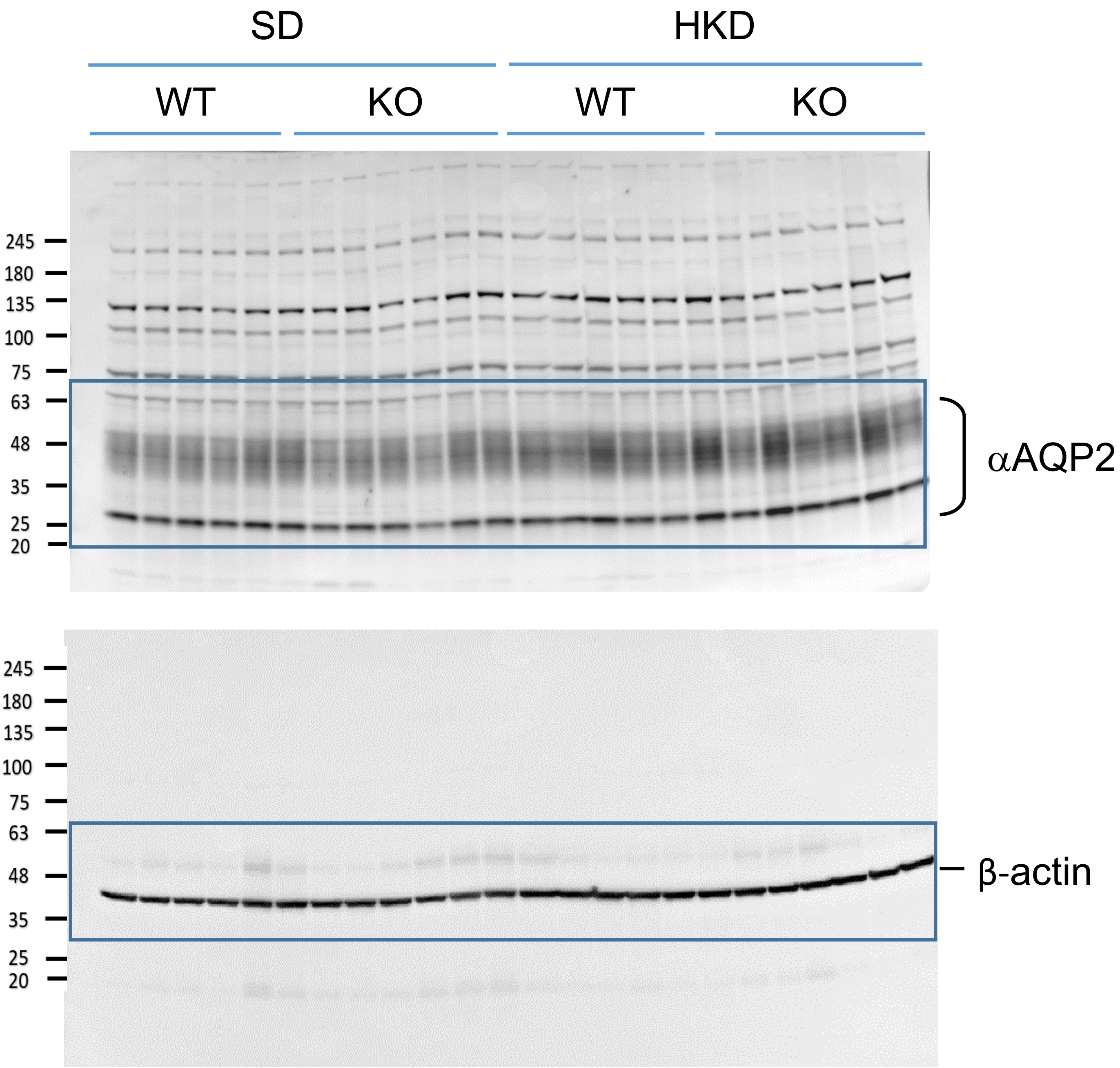

Supplement: Supplementary file 1 — Supplementary Material 1 [file 41598_2025_11106_MOESM1_ESM.pdf]
